# The CXCR3-CXCL11 signaling axis mediates macrophage recruitment and dissemination of mycobacterial infection

**DOI:** 10.1242/dmm.017756

**Published:** 2015-01-08

**Authors:** Vincenzo Torraca, Chao Cui, Ralf Boland, Jan-Paul Bebelman, Astrid M. van der Sar, Martine J. Smit, Marco Siderius, Herman P. Spaink, Annemarie H. Meijer

**Affiliations:** 1 Institute of Biology, Leiden University, Einsteinweg 55, 2333 CC, Leiden, The Netherlands; 2 Amsterdam Institute for Molecules, Medicines and Systems, Division Medicinal Chemistry, Faculty of Sciences, VU University, De Boelelaan 1105, 1081 HV, Amsterdam, The Netherlands; 3 Department of Medical Microbiology and Infection Control, VU University Medical Center, PO Box 7057, 1007 MB, Amsterdam, The Netherlands

**Keywords:** Macrophage biology, Tuberculosis, Chemokine, CXCR3, CXCL11, Mycobacterium, Zebrafish, Immunology

## Abstract

The recruitment of leukocytes to infectious foci depends strongly on the local release of chemoattractant mediators. The human CXC chemokine receptor 3 (CXCR3) is an important node in the chemokine signaling network and is expressed by multiple leukocyte lineages, including T cells and macrophages. The ligands of this receptor originate from an ancestral *CXCL11* gene in early vertebrates. Here, we used the optically accessible zebrafish embryo model to explore the function of the CXCR3-CXCL11 axis in macrophage recruitment and show that disruption of this axis increases the resistance to mycobacterial infection. In a mutant of the zebrafish ortholog of CXCR3 (*cxcr3.2*), macrophage chemotaxis to bacterial infections was attenuated, although migration to infection-independent stimuli was unaffected. Additionally, attenuation of macrophage recruitment to infection could be mimicked by treatment with NBI74330, a high-affinity antagonist of CXCR3. We identified two infection-inducible CXCL11-like chemokines as the functional ligands of Cxcr3.2, showing that the recombinant proteins exerted a Cxcr3.2-dependent chemoattraction when locally administrated *in vivo*. During infection of zebrafish embryos with *Mycobacterium marinum*, a well-established model for tuberculosis, we found that Cxcr3.2 deficiency limited the macrophage-mediated dissemination of mycobacteria. Furthermore, the loss of Cxcr3.2 function attenuated the formation of granulomatous lesions, the typical histopathological features of tuberculosis, and led to a reduction in the total bacterial burden. Prevention of mycobacterial dissemination by targeting the CXCR3 pathway, therefore, might represent a host-directed therapeutic strategy for treatment of tuberculosis. The demonstration of a conserved CXCR3-CXCL11 signaling axis in zebrafish extends the translational applicability of this model for studying diseases involving the innate immune system.

## INTRODUCTION

Macrophages are extremely dynamic phagocytic cells, able to integrate and respond to a wide spectrum of signals from infected tissues. A variety of receptors on their cell membrane can sense pathogen-associated molecular patterns (PAMPs), which induce the innate immune response (Medzhitov and Janeway, 2000). Some of these PAMPs, such as N-formylated bacterial peptides, have direct chemoattractant activity on phagocytes (Schiffmann et al., 1975). Moreover, a crucial contribution to efficient phagocyte recruitment is provided by lipidic and peptidic chemoattractant factors, produced or activated directly by the host locally at the infection site (Ford-Hutchinson et al., 1980; Lira and Furtado, 2012; Sun and Ye, 2012). In this group of compounds, the inflammatory chemokines play a major role. This subclass of small chemotactic proteins is induced upon infection and is able to exert target-specific activities towards subsets of leukocytes, both myeloid and lymphoid (Groom and Luster, 2011). In humans, CXCL9 [also known as MIG (monokine-induced by IFN-γ)], CXCL10 [IP-10 (IFN-γ-inducible protein 10)] and CXCL11 [I-TAC (T cell α chemoattractant)] are IFN-inducible chemokines and mediate recruitment of T cells, natural killer (NK) cells and monocytes/macrophages at the infection site, predominantly through their cognate G-protein coupled receptor, CXCR3 (Janatpour et al., 2001; Loetscher et al., 1996). This signaling axis has been implicated in several physiological activities, including maturation of T cells and vasculogenesis (Liu et al., 2005; Zhou et al., 2010). Additionally, CXCR3 and its ligands have been linked to inflammatory and immune-related diseases, of autoimmune (Bondar et al., 2014; Lacotte et al., 2009; Liu et al., 2005; Müller et al., 2010), infectious (Chakravarty et al., 2007; Cohen et al., 2013; Rosas et al., 2005; Seiler et al., 2003) or malignant (Fulton, 2009; Kawada et al., 2007; Oghumu et al., 2014; Pan et al., 2006) nature. Most of the literature on mammalian systems focuses on the role of this receptor in maturation, priming, activation and migration of T cells (Bondar et al., 2014; Liu et al., 2005; Slütter et al., 2013). However, recent studies have demonstrated that CXCR3 also plays an important role in directing macrophage activities, both under physiological and under pathological conditions (Cuenca et al., 2011; Kakuta et al., 2012; Oghumu et al., 2014; Zhou et al., 2010).

The zebrafish embryo model provides a useful platform to study chemokine-dependent cell migration, combining excellent possibilities for intravital imaging with the availability of a vast array of genetic tools (Raz and Mahabaleshwar, 2009). Homologous relationships between mammalian and zebrafish CXCR4-CXCL12 and CXCR2-CXCL8 receptor-ligand pairs have been well established and studies in zebrafish have contributed significantly to the understanding of the role of these signaling axes in developmental processes, neutrophil motility, long-range neutrophil mobilization and infection-induced chemotaxis (David et al., 2002; Deng et al., 2013; Doitsidou et al., 2002; Sarris et al., 2012; Walters et al., 2010). Based on phylogeny reconstructions, the CXCR3-CXCL11 axis emerged for the first time in a common ancestor of zebrafish and mammals (Xu et al., 2014). In placental mammals, amphibians and reptiles, a single copy per haplotype of *CXCR3* is generally present, whereas *CXCR3* was lost in the divergence of avian and marsupial mammalian clades. Several teleost fish show an expansion of the CXCR3 family (Aghaallaei et al., 2010; Chang et al., 2007; Xu et al., 2014), including zebrafish, where three copies, namely *cxcr3.1* (ENSDARG00000007358), *cxcr3.2* (ENSDARG00000041041) and *cxcr3.3* (ENSDARG00000070669), are located in tandem on chromosome 16 (Nomiyama et al., 2013). The *CXCL9-CXCL10-CXCL11* triplet of CXCR3 ligands in mammals is likely to have originated from a relatively recent common ancestor (O’Donovan et al., 1999). The situation in fish is variegated and, in some cases, specific expansions have taken place. In zebrafish, a cluster of seven putative *cxcl11* genes, which are grouped together in a single locus on chromosome 5, share both homology and synteny with human *CXCL11* (Nomiyama et al., 2013). However, an association between the different isoforms of Cxcl11 ligands and Cxcr3 receptors has not been described, and the *in vivo* relevance of this signaling axis in the zebrafish model has not been addressed.

**TRANSLATIONAL IMPACT**

**Clinical issue**

Mycobacteria are the causative agents of chronic, life-threatening infectious diseases such as tuberculosis and leprosy. In order to replicate and spread within their host, mycobacteria highjack one of the primary immune defense cells: the macrophage. Recruitment of macrophages relies heavily on the production of chemokines by the infected host. However, the role of chemokine signaling in mycobacterial disease remains poorly explored. CXC chemokine receptor 3 (CXCR3) is an important node in the chemokine signaling network and has been extensively studied in T cells. Emerging evidence suggests that CXCR3 also has important functions in macrophages that might be linked with immune-related diseases.

**Results**

In this study, the authors used a zebrafish model of tuberculosis to investigate the role of CXCR3 in macrophages during the early stages of mycobacterial infection. They found that mutation of a zebrafish CXCR3 homolog attenuates the infection-dependent recruitment of macrophages and limits the dissemination of the pathogen via macrophage carriers. This results in a reduced formation of granulomatous lesions, typical of mycobacterial disease. Similar attenuation of macrophage attraction to local infections could be achieved by treatment with NBI74330, a high-affinity antagonist of CXCR3. The authors also purified the zebrafish counterparts of the human chemokine (C-X-C motif) ligand 11 (CXCL11) family and demonstrated that two of these are inducible by infection and specifically recruit macrophages via the CXCR3 receptor in the zebrafish model.

**Implications and future directions**

This study is the first to implicate the CXCR3-CXCL11 signaling axis in macrophage responses that drive the initiation and expansion of mycobacterial granulomas, the pathological hallmark of tuberculosis disease. The beneficial effect of CXCR3 mutation on the control of mycobacterial infection in the zebrafish host should drive further research into the CXCR3-CXCL11 axis as a potential target for host-directed therapy against tuberculosis. Research into such novel therapeutic approaches is important in view of the increasing prevalence of antibiotic-resistant mycobacterial strains. Defects in CXCR3 signaling have also been associated with other immune-related diseases, including cancer and inflammatory disorders. Therefore, the finding that the CXCR3-CXCL11 axis and its sensitivity to pharmacological inhibition are conserved between human and zebrafish has broad implications for the translational value of this model.

In previous work we have shown that one of the three *CXCR3* paralogs, *cxcr3.2*, is expressed in macrophages of 1-day-old zebrafish embryos (Zakrzewska et al., 2010). In the present study we used a *cxcr3.2* mutant to investigate the role of Cxcr3 signaling in macrophage mobilization and function. In agreement with previous morpholino knockdown results, the receptor loss-of-function resulted in the attenuation of macrophage recruitment to local infection with *Salmonella typhimurium*. Moreover, we identified two infection-inducible CXCL11-like chemokines, which act as functional ligands of Cxcr3.2 with chemoattractant activity on macrophages. Finally, we demonstrate here that *cxcr3.2* is required for efficient recruitment of macrophages to *Mycobacterium marinum* infection and for the dissemination of this pathogen into host tissues, which is driven by macrophages. The zebrafish-*M. marinum* host-pathogen pair is widely used to model human tuberculosis and has provided important insights into the interaction of mycobacteria with host macrophages (Cambier et al., 2014; Clay et al., 2007; Davis et al., 2002; Roca and Ramakrishnan, 2013; Torraca et al., 2014; van der Vaart et al., 2014). *M. marinum* is closely related to the human pathogen *Mycobacterium tuberculosis*, and the zebrafish model replicates the formation of granulomas, the typical histopathological hallmark of human tuberculosis (Cronan and Tobin, 2014; Ramakrishnan, 2013). The results presented here demonstrate a novel function for the CXCR3-CXCL11 signaling axis in macrophage responses that drive the initiation and expansion of these granulomatous lesions that are crucial for the dissemination of mycobacterial infection.

## RESULTS

### *cxcr3.2* is expressed in phagocyte populations during zebrafish embryonic and larval development

**Fig. 1.**
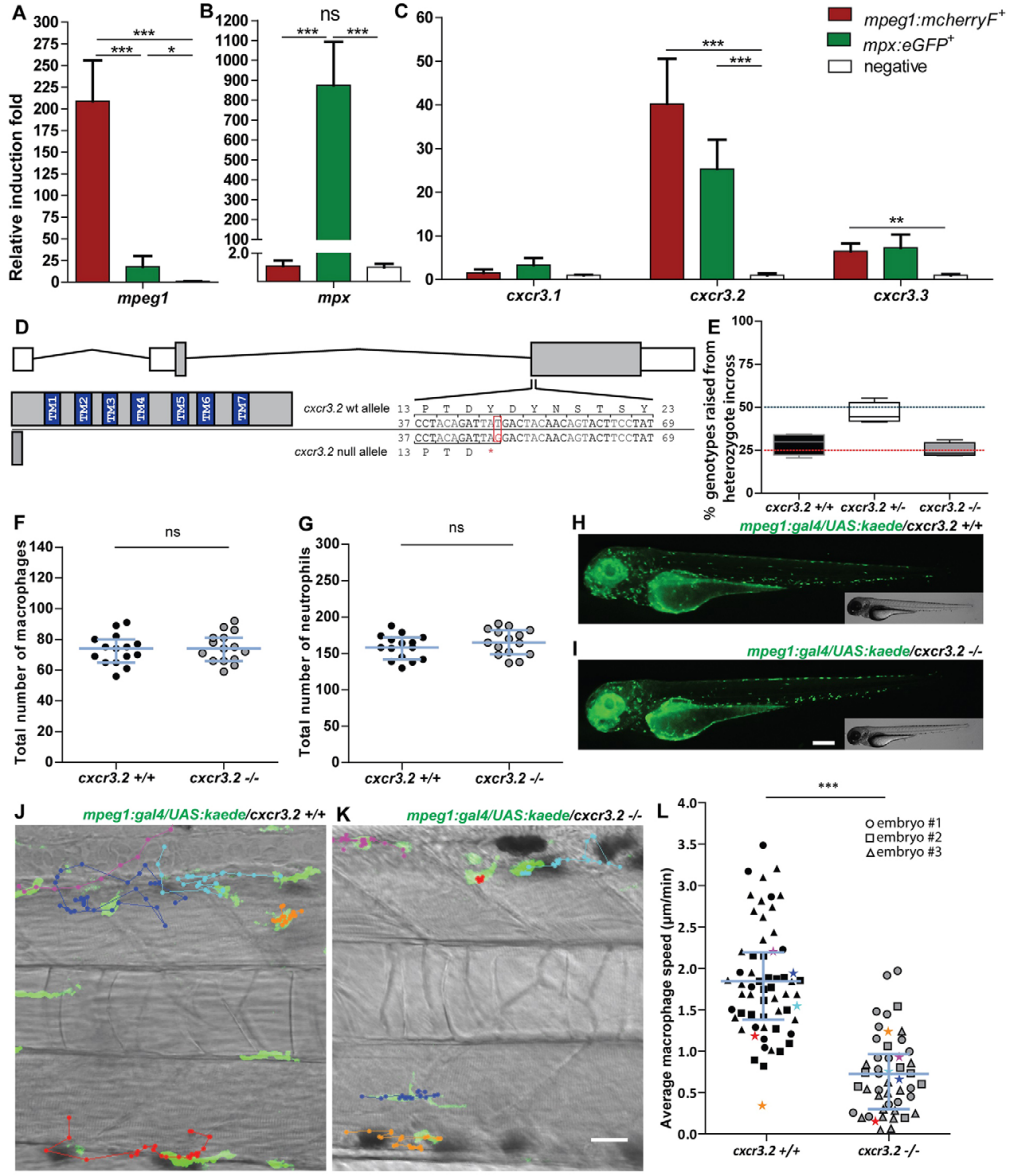
**Characterization of *cxcr3.2*^−/−^ embryos in unchallenged conditions.** (A–C) Expression of *cxcr3.2* and its paralogs *cxcr3.1* and *cxcr3.3* in FACS-sorted phagocytes. Graphs represent the relative induction fold of the macrophage marker *mpeg1* (A), the neutrophil marker *mpx* (B), and of the *cxcr3* paralogs (C) in FACS-sorted macrophages and neutrophils from the combined transgenic line *Tg(mpeg1:mcherryF/mpx:eGFP)* at 2 dpf. Expression of *cxcr3.2* and *cxcr3.3* could be detected in both macrophages and neutrophils, whereas *cxcr3.1* was not significantly enriched in the FACS-sorted populations when compared with the non-labeled cell fraction. Sample size (*n*): five replicates. Errors bars: mean±s.e.m. Reference gene: *eif4a1b*. (D) Effect of the *cxcr3.2* point mutation. Top: gene structure of *cxcr3.2*. Boxes represent exons, of which the gray parts correspond to the coding sequence. Bottom right: *cxcr3.2* wild-type (wt) and mutant allele. A single T-to-G mutation at nucleotide 48 generates an early stop codon. Bottom left: consequence of *cxcr3.2* mutation at the protein level. In *cxcr3.2* mutant zebrafish, only a peptide of 15 amino acids can be translated, which lacks all the conserved transmembrane domains (TM1-7). Nucleotide and amino acid positions are enumerated from the translation start codon. (E) Normal viability of *cxcr3.2^−/−^* mutants. Percentage of genotypes deriving from *cxcr3.2^+/−^* incross. No significant deviation from the Mendelian 1:2:1 ratio was observed. The genotypes were evaluated on 122 adult fish from four independent breedings. The boxplots represent the area of the distributions between the first and the third quartiles. Whiskers represent the minimum and maximum end points of the distributions. (F,G) Quantification of macrophages and neutrophils. Combined Leukocyte-plastin (Lp) immunostaining and Myeloperoxidase (Mpx) staining were performed on *cxcr3.2*^+/+^ and *cxcr3.2*^−/−^ embryos at 3 dpf and the numbers of stained cells residing in the caudal hematopoietic tissue were counted. Exclusively Lp-stained cells were considered as macrophages (F) and Lp/Mpx double-positive cells as neutrophils (G). No significant differences were detected. Total number of larvae (*n*) per group in both F and G: 15. Error bars: median and interquartile range. (H,I) Spatial distribution of macrophages. A macrophage-specific transgenic reporter driven by the *mpeg1* promoter [*Tg(mpeg1:gal4/UAS:kaede)* (Ellett et al., 2011)] was crossed into the *cxcr3.2* mutant background. Representative images of the resulting *Tg(mpeg1:gal4/UAS:kaede) cxcr3.2^+/+^* (H) and *cxcr3.2^−/−^* (I) larvae at 3 dpf show no major differences in the macrophage distribution pattern. Scale bar: 200 μm. (J,K) Macrophage basal migratory capability. Paths of five representative macrophages in the trunk of *Tg(mpeg1:gal4/UAS:kaede) cxcr3.2^+/+^* (J) and *cxcr3.2*^−/−^ (K) larvae at 3 dpf. Mutant and wt larvae were mounted in agarose on the same dish and behavior of mutant and wt macrophages were simultaneously followed for 3 hours. Time-lapse images were taken every 6 minutes. The paths were followed and analyzed using ImageJ ManualTrack plugin. See also supplementary material Movies 1, 2. Scale bar: 20 μm. (L) Quantification of basal migration difference. The average speed of individual macrophages was calculated by tracking 15–21 macrophages from three different *Tg(mpeg1:gal4/UAS:kaede) cxcr3.2^+/+^* and *cxcr3.2*^−/−^ larvae (each larva is indicated with a different symbol) and was significantly reduced in *cxcr3.2^−/−^* macrophages. Total number of tracks (*n*): 61, 48. Error bars: mean and interquartile range. ns, non-significant; *P<0.05; ***P<0.001.

We previously reported that *cxcr3.2* expression could be detected by fluorescent *in situ* hybridization at 1 day post fertilization (dpf) in phagocytes expressing the macrophage marker *csf1r* (*colony stimulating factor 1 receptor*) and not in cells positive for the neutrophil marker *mpx* (*myeloperoxidase*) (Zakrzewska et al., 2010). However, we were unable to detect its expression with the same method at later stages. To determine whether *cxcr3.2* continues to be expressed in macrophages during the embryonic and larval development, we analyzed RNA expression levels from FACS-sorted *mpeg1:mcherryF*^+^ and *mpx:eGFP^+^* cells from the double-transgenic line *Tg(mpeg1:mcherryF/mpx:eGFP)*. These data show that macrophages (*mpeg1:mcherryF^+^* population) maintain *cxcr3.2* expression at 2 and at 6 dpf (Fig. 1A–C and supplementary material Fig. S1). Expression of *cxcr3.2* could also be detected in neutrophils (*mpx:eGFP^+^* population). In addition, *cxcr3.3* could be detected in both phagocyte types, whereas *cxcr3.1* was not specifically enriched in the sorted cell populations (Fig. 1C and supplementary material Fig. S1).

### *The cxcr3.2^hu6044^* line carries a nonsense mutation in *cxcr3.2*

Sequencing of an ENU (N-ethyl-N-nitrosourea)-mutagenized zebrafish library resulted in the identification of a *cxcr3.2* mutant allele, *cxcr3.2^hu6044^*, which carries a T-to-G (deoxythymidine to deoxyguanosine) substitution, creating a premature stop codon. This mutation leads to the interruption of the protein translation after 15 amino acids, before the region that encodes all the transmembrane domains that are essential for the function of the receptor (Fig. 1D and supplementary material Fig. S2). The nonsense *cxcr3.2^hu6044^* mutation is not likely to lead to a functional truncated protein by using a downstream AUG codon as a signal for translation initiation. The second AUG in frame is located 354 nucleotides (118 amino acid residues) downstream from the canonical start codon and use of this codon as a translation start would lead to a truncated product lacking both the most N-terminal extracellular domain and the first two transmembrane domains. Furthermore, because the mutation occurs downstream of all the splicing sites, the possibility of alternative splicing and/or altered pre-RNA maturations seems unlikely and this was excluded by sequencing of the cDNA of *cxcr3.2* in mutants and wild types (wt). The *cxcr3.2* locus is closely linked to the loci of *cxcr3.1* and *cxcr3.3* owing to their chromosome proximity. To evaluate the presence of additional alterations in these genes as a consequence of the ENU mutagenesis, we sequenced their genetic loci in the AB/TL wt strain in our facility (used to outcross the mutant) and in two families of *cxcr3.2^+/+^* and *cxcr3.2*^−/−^ fish. We did not identify additional nonsense mutations, although we could detect several non-synonymous single-nucleotide polymorphisms (nsSNPs), which are described in supplementary material Table S1. However, all the nsSNPs that were found in the *cxcr3.2^−/−^* line were also present in the AB/TL fish line, indicating that these changes are likely to be natural wt polymorphisms and not an effect of the ENU mutagenesis. To address the possible relevance of these nsSNPs with respect to the protein function, we used the PROVEAN software tool (Protein Variation Effect Analyzer; http://provean.jcvi.org) (Kumar et al., 2009; Choi et al., 2012). None of the nsSNPs was predicted to impact on the protein functionality (supplementary material Table S1). Therefore, it is unlikely that single amino acid replacements in the protein sequences of Cxcr3.1 or Cxcr3.3 would affect the phenotype of fish carrying the *cxcr3.2^hu6044^* nonsense allele.

### *cxcr3.2* mutation does not affect macrophage development but alters their basal motility

Crossing of heterozygous *cxcr3.2^hu6044^* carriers generated homozygous embryos in Mendelian proportions with their heterozygous and wt siblings. Screening of the adult offspring from a heterozygous incross showed that, in laboratory conditions, *cxcr3.2^−/−^* fish do not exhibit differences in survival and development compared with their wt siblings (Fig. 1E) and could only be distinguished by genotyping.

Based on combined immunostaining for the pan-leukocyte marker Leukocyte-plastin (Lp) and Mpx activity staining (Cui et al., 2011), *cxcr3.2^−/−^* embryos showed similar numbers of macrophages and neutrophils as their wt siblings (Fig. 1F,G). With the aim of investigating the relevance of *cxcr3.2* expression in macrophage behavior, we crossed the *cxcr3.2* mutation into the *Tg(mpeg1:gal4/UAS:kaede)* background. Labeled macrophages showed similar numbers and spatial distribution in mutant and wt (Fig. 1H,I). However, a basal macrophage migratory deficiency was observed in the mutants (Fig. 1J–L and supplementary material Movies 1, 2). The aberrant motility might be explained by the presence of constitutive quantities of Cxcr3.2 ligands in the macrophage microenvironment, which could contribute to a higher basal activity of *cxcr3.2^+/+^* macrophages.

### Mutation of *cxcr3.2* does not affect chemoattraction of macrophages by *Cxcr3.2*-independent factors

**Fig. 2.**
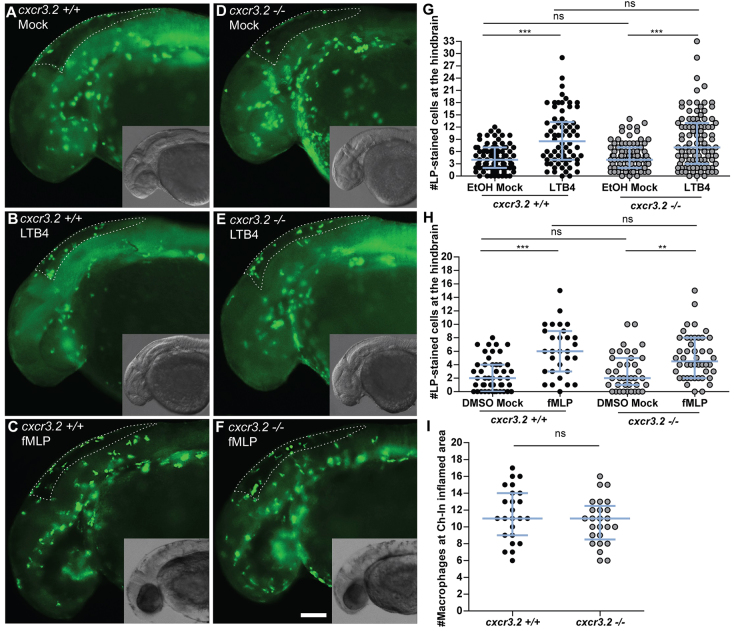
**Macrophage chemoattraction by Cxcr3.2-independent factors.** (A–H) Chemoattraction of macrophages by LTB_4_ and fMLP. *cxcr3.2*^+/+^ (A–C) and *cxcr3.2*^−/−^ (D–F) embryos at 30 hpf were locally injected into the hindbrain cavity with 10.1 ng/ml (30 nM) LTB_4_ (representative images B and E) or with 0.2 mg/ml (0.5 mM) of fMLP (representative images C and F). Mock control injections with the solvents were 0.02% EtOH in PBS for the LTB_4_ treatment and 5% DMSO in PBS for the fMLP treatment (representative images A and D). Lp-stained cells accumulated in 3 hours within the hindbrain limits (dotted line) were counted as macrophages, as neutrophils do not significantly contribute to the total number of leukocytes recruited to the hindbrain at this developmental stage (supplementary material Fig. S3). LTB_4_ and fMLP stimulation resulted in significantly increased macrophage recruitment, compared with the mock injection, and this was independent of *cxcr3.2* mutation (G,H). Scale bar in A–F: 100 μm. Data were accumulated from three independent experiments. Sample size (*n*) in G: 82, 70, 86, 100; Sample size (*n*) in H: 47, 30, 39, 46. Error bars: median and interquartile range. (I) Local macrophage recruitment to CuSO_4_ chemically-induced inflammation. Copper sulphate treatment was performed on 3 dpf embryos and macrophages accumulated in 3 hours at the damaged neuromasts of the lateral line were counted as Lp-stained cells minus Lp/Mpx double-positive cells, representing neutrophils. Similar numbers of macrophages were recruited to inflamed areas in both *cxcr3.2^+/+^* and *cxcr3.2^−/−^* embryos. Sample size (*n*): 24, 25. Error bars: median and interquartile range. ns, non-significant; **P<0.01; ***P<0.001.

To test whether the basal motility defect of *cxcr3.2^−/−^* macrophages affected the stimulus-directed chemoattraction to *cxcr3.2*-independent factors, we locally injected the chemoattractant factors leukotriene B4 (LTB_4_) and N-formyl-methionyl-leucyl-phenylalanine peptide (fMLP) into the hindbrain ventricle of embryos at 30 hpf (hours post fertilization). At this developmental stage, the neutrophil population is not fully differentiated (Henry et al., 2013; Lieschke et al., 2001) and the population of phagocytes infiltrating the hindbrain upon chemotactic stimulation consists predominantly of macrophages (supplementary material Fig. S3); therefore, we could use Lp immunostaining to identify recruited macrophages. No significant difference was observed in the numbers of *cxcr3.2^+/+^* and *cxcr3.2^−/−^* macrophages accumulated to either stimuli in 3 hours (Fig. 2A–H). In addition, we also employed a previously described chemically induced inflammation (ChIn) assay (d’Alençon et al., 2010), using copper sulphate treatment of embryos at 3 dpf to induce acute inflammation of lateral line neuromast hair cells. At this stage, macrophages were counted as Lp-positive and Mpx-negative cells using the combined Lp/Mpx staining. Also in this assay, no significant difference in the numbers of macrophages recruited to the inflamed neuromasts was observed between *cxcr3.2^+/+^* and *cxcr3.2^−/−^* larvae (Fig. 2I). Therefore, we concluded that *cxcr3.2* mutation does not affect the capability of macrophages to respond to stimulatory sources independent of Cxcr3.2 signaling and that the basal motility defect does not influence the experimentally induced macrophage recruitment.

### Early migration of macrophages to localized infection is affected by mutation of *cxcr3.2* or treatment with a CXCR3 antagonist

**Fig. 3.**
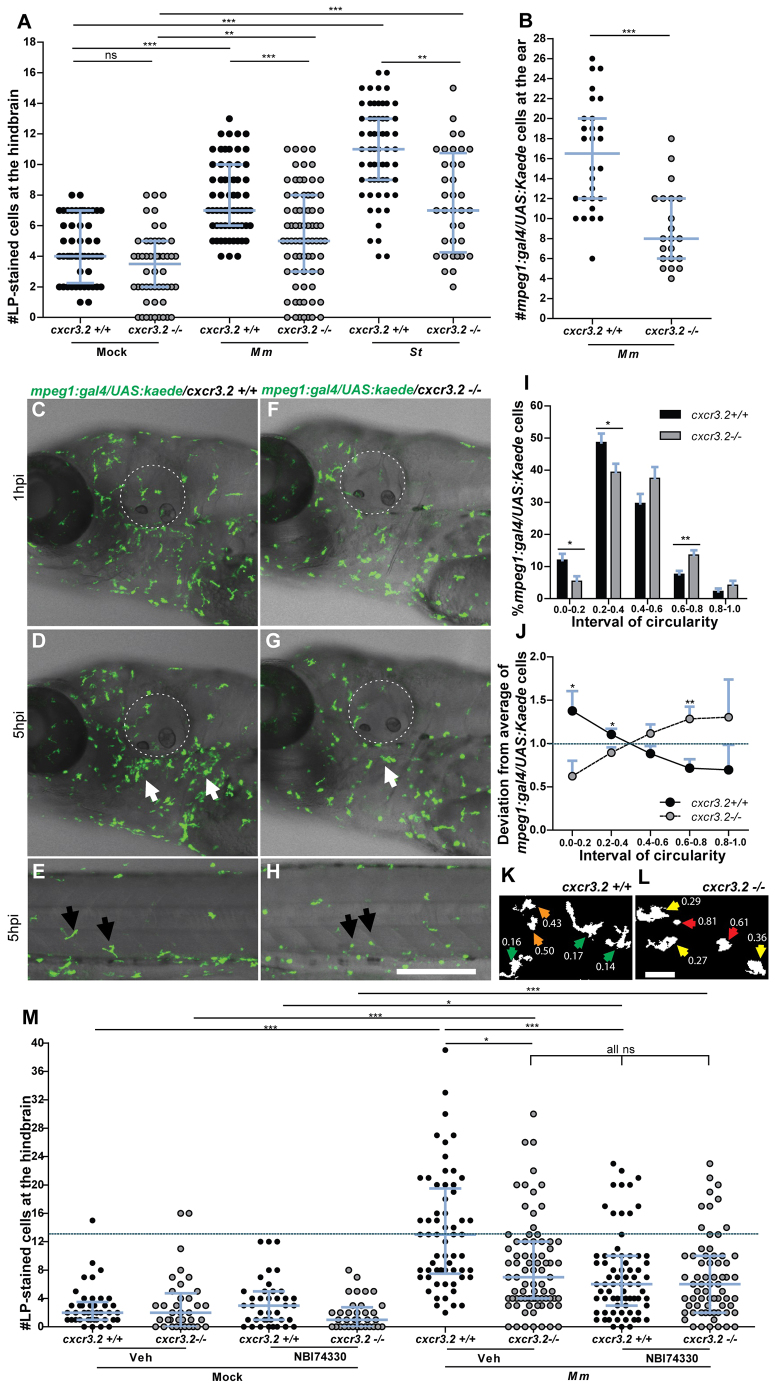
**Cxcr3.2-dependent macrophage recruitment to localized bacterial infections.** (A) *M. marinum* and *S. typhimurium* infection in the hindbrain ventricle. *cxcr3.2*^+/+^ and *cxcr3.2*^−/−^ embryos were injected at 30 hpf with 200 CFU of *M. marinum* (*Mm*) or *S. typhimurium* (*St*), or mock injected with 2% polyvinylpyrrolidone-40 in PBS. Lp-stained cells accumulated in 3 hours within the hindbrain limits were counted as macrophages, because neutrophils do not significantly contribute to the total number of leukocytes recruited to the hindbrain at this developmental stage (supplementary material Fig. S3). Significant reduction of macrophage chemotaxis to infection is determined by *cxcr3.2* mutation. Data were accumulated from two (*St*) or three (*Mm*) independent experiments. Sample size (*n*): 52, 52, 63, 75, 60, 36. Error bars: median and interquartile range. (B–H) Macrophage recruitment and systemic activation following *M. marinum* infection in the otic vesicle. *Tg(mpeg1:gal4/UAS:kaede) cxcr3.2^+/+^* and *Tg(mpeg1:gal4/UAS:kaede) cxcr3.2^−/−^* larvae were injected with 200 CFU of *M. marinum* into the otic vesicle (dotted line) at 3 (B) or 4 (C–H) dpf. At 4 hpi of 3 dpf larvae, the accumulation of *mpeg1:gal4/UAS:kaede*-positive cells within the perimeter of the otic vesicle was reduced in *cxcr3.2*^−/−^ larvae (B). Following injection at 4 dpf, macrophages are less able to penetrate the otic vesicle, but accumulate in the surrounding area (white arrows in D and G), as shown in representative time course movies (supplementary material Movies 3, 4) and stills from these movies at 1 hpi (C,F) and 5 hpi (D,G). A reduced accumulation was observed in the *cxcr3.2* mutant. At a distal location in the trunk (E,H), macrophages seemed to show more frequently a branched morphology in *cxcr3.2^+/+^* and a round morphology in *cxcr3.2^−/−^* (black arrows in E and H). Sample size (*n*) in B: 28, 21. Error bars: median and interquartile range. Scale bar in C–H: 250 μm. (I–L) Quantification of distal macrophage activation following local *M. marinum* infection. In order to quantify the distal activation of macrophages upon *M. marinum* infection, 200 CFU of bacteria were injected in the otic vesicle of 3 dpf *mpeg1:gal4/UAS:kaede* larvae. Images of the macrophages were acquired from the trunk of the infected larvae at 4 hpi and the circularity index (CI) of the distal macrophages was calculated. The graph in I represents the percentage of macrophages residing in the different intervals of CI, whereas the graph in J represents the divergence in distribution of the *cxcr3.2^+/+^* and *cxcr3.2^−/−^* macrophages in the different classes of CI, calculated as the ratio between the percentage of *cxcr3.2^+/+^* or *cxcr3.2^−/−^* macrophages in a certain CI interval and the overall mean percentage [(mutant + wt)/2] of macrophages in that interval (see Materials and Methods). Macrophages of *cxcr3.2^+/+^* and *cxcr3.2^−/−^* larvae were distributed in a different fashion along the different classes, with the classes of high circularity more populated in the mutants and the classes of reduced circularity more populated in the wild type (wt). K and L show representative macrophages analyzed in I and J and their corresponding CI. Green arrows: 0.0≤CI<0.2; yellow arrows: 0.2≤CI<0.4; orange arrows: 0.4≤CI<0.6; red arrows: 0.6≤CI≤1.0. Sample size (*n*) in I–L: 390 *cxcr3.2^+/+^* and 293 *cxcr3.2^−/−^* cells from 14 and 16 embryos, respectively. Error bars: mean±s.e.m. Scale bar in K,L: 40 μm. (M) Attenuation of macrophage recruitment to *M. marinum* via treatment with the CXCR3 antagonist NBI74330. *cxcr3.2*^+/+^ and *cxcr3.2*^−/−^ embryos were bath-exposed to 50 μM NBI74330 or to vehicle only (Veh; 0.5% DMSO in medium) for 3 hours and then injected in the hindbrain ventricle at 30 hpf with mock or 200 CFU of *Mm.* Embryos were kept in NBI74330 or vehicle medium for an additional 3 hours and then collected for Lp immunostaining. Treatment with NBI74330 reduced the macrophage chemotaxis to infection in *cxcr3.2^+/+^* embryos to similar levels as the vehicle-treated *cxcr3.2^−/−^* embryos, and no significant additive effect of *cxcr3.2* mutation and NBI74330 treatment was observed. Data were accumulated from two independent experiments. Sample size (*n*): 41, 36, 39, 36, 61, 79, 73, 67. Error bars: median and interquartile range. ns, non-significant; *P<0.05; **P<0.01; ***P<0.001.

To determine whether Cxcr3.2 signaling contributes significantly to the recruitment of macrophages to different types of bacterial infections, we injected either *M. marinum* or *S. typhimurium* into the hindbrain ventricle of embryos at 30 hpf. In both infection models, a significant reduction of the number of macrophages accumulating at the infected site was detected in *cxcr3.2^−/−^* embryos at 3 hpi (hours post injection) (Fig. 3A). A similar reduction of macrophage recruitment was also observed when *M. marinum* was locally injected into the otic vesicle at 3 dpf (Fig. 3B). To visualize the dynamics of the macrophage migration *in vivo*, we used the combined mutant-transgenic line *Tg(mpeg1:gal4/UAS:kaede)/cxcr3.2^−/−^* and followed the early response of *mpeg1*-positive cells to *M. marinum* infection in the otic vesicle of 4 dpf larvae by confocal time-lapse imaging. In agreement with the previous results, a difference in the trend of macrophage recruitment was observed between the mutant and the wt over a time course of 5 hours (Fig. 3C–H; supplementary material Movies 3, 4). Furthermore, at locations distal from the infection site, macrophages in wt larvae showed more frequently an activated morphology with formation of branched protrusions (Fig. 3E) when compared with the mutant line (Fig. 3H). To quantify this phenomenon, we classified the distal macrophages of locally infected larvae according to their circularity index (CI), which estimates by an index between 0 and 1 the level of divergence of the cell shape projection from a perfect circle (CI=1). The different intervals of circularity were differently populated in wt and mutant larvae, with the classes of reduced circularity (0 to 0.4) being more populated in *cxcr3.2^+/+^* larvae and the classes of higher circularity (0.6–0.8) being more populated in *cxcr3*.2^−/−^ larvae (Fig. 3I–L). These results provide evidence that the Cxcr3.2-dependent signaling pathway mediates a significant component of the macrophage recruitment to pathogens in the early phase of the infection. To determine whether the infection-dependent macrophage recruitment can also be modulated pharmacologically, we tested a chemical inhibitor of human CXCR3, NBI74330 (Scholten et al., 2014), which binds with high affinity to a pocket formed by the transmembrane domains of CXCR3. Key amino acid residues in this pocket are conserved between the human and the zebrafish receptors (supplementary material Fig. S4). Treatment with this CXCR3 antagonist attenuated the macrophage recruitment to local *M. marinum* infection in *cxcr3.2^+/+^* embryos to a similar level as that of the vehicle-treated *cxcr3.2^−/−^* embryos and did not show a cooperative effect with the *cxcr3.2* mutation (Fig. 3M). These results support the conservation of CXCR3 signaling between fish and mammals.

### A group of CXCL11-like chemokines are inducible upon local and systemic infection in zebrafish

**Fig. 4.**
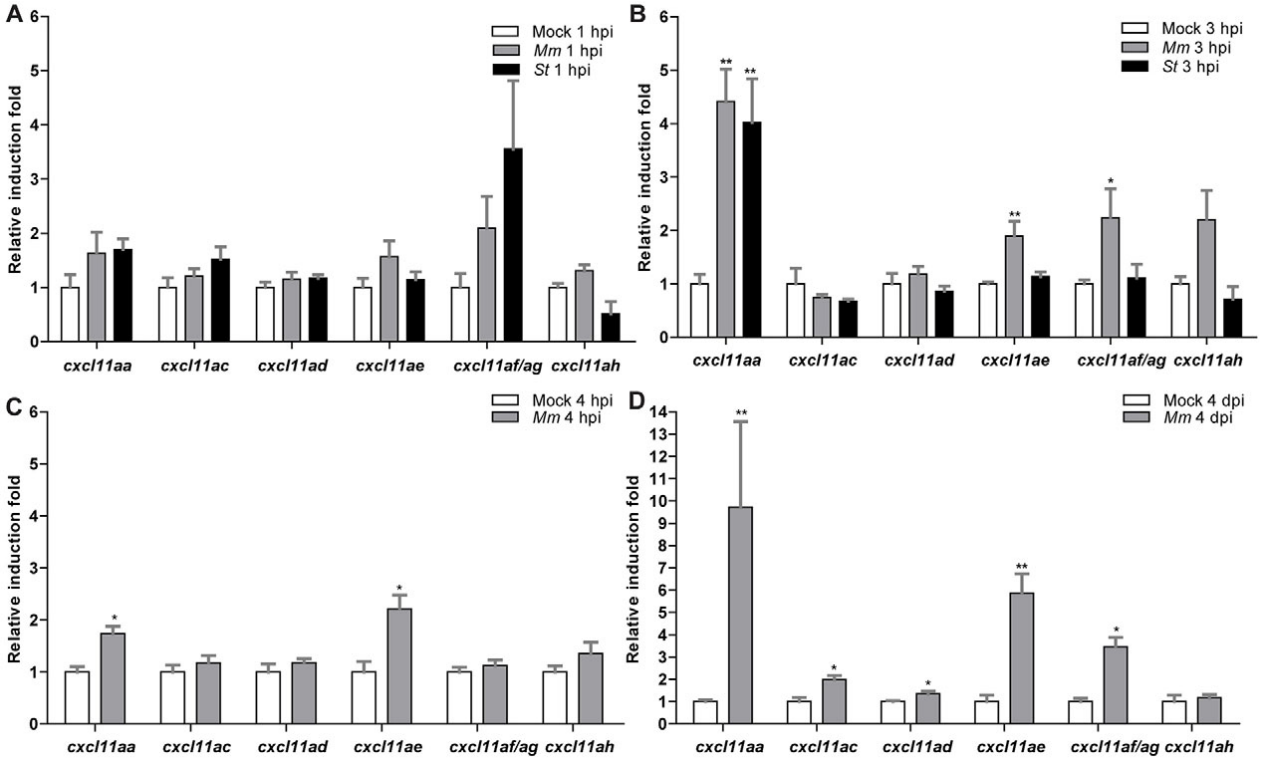
**Inducibility of a subset of *cxcl11-like* genes upon bacterial infection.** (A,B) Quadruplicate pools of 18–20 embryos infected in the hindbrain with 200 CFU of *M. marinum* (*Mm*) or *S. typhimurium* (*St*) at 30 hpf or mock-injected with PBS were collected at 1 hpi (A) and 3 hpi (B). A subset of *CXCL11*-like chemokine genes shows upregulation by qPCR in the infected groups, which becomes significant at 3 hpi. Error bars: mean±s.e.m. (C,D) Triplicate pools of 18–20 embryos systemically infected with *M. marinum* (*Mm*) at 1 dpf or mock-injected with PBS were collected at 4 hpi (C) and 4 dpi (D). Expression of *cxcl11aa* and *cxcl11ae* was significantly induced at both time points, whereas *cxcl11ac*, *cxcl11ad* and *cxcl11af/ag* were significantly induced only at 4 dpi. Error bars: mean±s.e.m. *P<0.05; **P<0.01.

Although our analysis of the *cxcr3.2* mutant supports the role of Cxcr3.2 in macrophage chemotaxis to infections, the chemokine ligands that signal via this receptor are unknown. The assignment of ligand-receptor pairs is complicated by the relatively poor conservation of chemokine sequences among vertebrates and the species-specific expansions of the chemokine gene clusters (Nomiyama et al., 2013). However, systematic study of the orthologous relationships between vertebrate chemokines indicated that seven *CXCL11*-like chemokine genes, located in tandem on chromosome 5 (*cxcl11aa*, *cxcl11ac*, *cxcl11ad*, *cxcl11ae*, *cxcl11af*, *cxcl11ag*, *cxcl11ah*), have evolved in zebrafish as a counterpart to the mammalian *CXCL9*, *CXCL10* and *CXCL11* genes (Nomiyama et al., 2013). The amino acid similarity between the CXCL11-like chemokines in zebrafish and human CXCL11 exceeds the similarity that human CXCL9, CXCL10 and CXCL11 show among each other (supplementary material Fig. S5; Table S2). We therefore considered the zebrafish CXCL11-like chemokines as putative ligands for the Cxcr3.2 receptor. Because Cxcr3.2 was clearly involved in the early phase of the infection response, we reasoned that the ligands that induce Cxcr3.2-mediated chemotaxis should be promptly upregulated upon local infection. For this reason, we collected RNA samples from whole embryos infected in the hindbrain with 200 colony-forming units (CFU) of either *S. typhimurium* or *M. marinum* at 1 and 3 hpi and designed gene-specific primers for the members of the *cxcl11* gene cluster. Because of the high level of sequence conservation between *cxcl11af* and *cxcl11ag* (only 2 bp difference on the cDNA leading to a single semiconservative residue change of an aspartic acid with a glutamic acid), a promiscuous primer pair was used that can amplify both gene transcripts (*cxcl11af/ag*). Analysis by qRT-PCR revealed that, at 1 hpi, *cxcl11af/ag* showed twofold upregulation with *M. marinum* infection and fourfold upregulation with *S. typhimurium* infection, although no statistical significance was observed compared with the mock-injected controls (Fig. 4A). At 3 hpi the expression of *cxcl11aa*, *cxcl11ae* and *cxcl11af/ag* was significantly upregulated to levels of two- to 4.5-fold (Fig. 4B). In particular, *cxcl11aa* displayed the highest levels of induced transcription (~4.5-fold induction) with both the pathogens tested, suggesting this chemokine as an effective signaling ligand of Cxcr3.2 involved in the response to infection. We verified that the genes induced by local infection were also responsive to systemic infection with *M. marinum*. Upregulation of *cxcl11aa* and *cxcl11ae* was detected both at 4 hpi and at 4 dpi (days post injection) during *M. marinum* systemic infection, whereas *cxcl11af/ag* was significantly induced to levels of ~fourfold only at the later stage of infection (Fig. 4C,D). Additionally, at this time point, *cxcl11ac* and *cxcl11ad* were also significantly upregulated to levels of 1.5- to two-fold (Fig. 4D).

### Recombinant Cxcl11aa and Cxcl11af exert macrophage chemoattraction *in vivo* in a Cxcr3.2-dependent manner

**Fig. 5.**
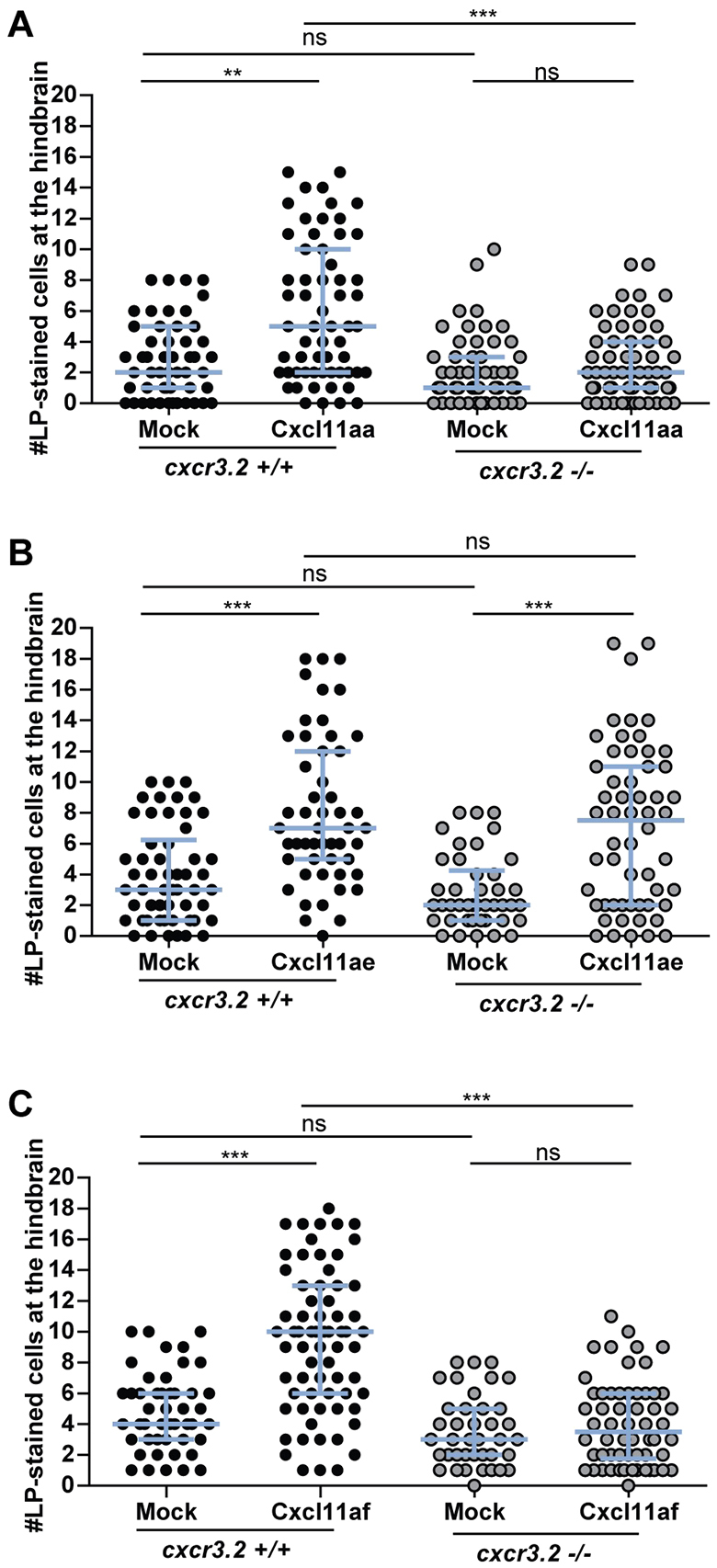
**Macrophage chemoattraction by locally injected recombinant chemokines in *cxcr3.2* mutant and wild-type siblings.** Recombinant proteins or buffer (mock) were injected into the hindbrain ventricle at 30 hpf and macrophages accumulating in 3 hours within the hindbrain limits were counted as Lp-stained cells. Data were accumulated from three independent experiments. (A) Cxcl11aa (1.2 mg/ml). Sample size (*n*): 51, 60, 55, 60. (B) Cxcl11ae (0.5 mg/ml). Sample size (*n*): 54, 51, 42, 56. (C) Cxcl11af (0.5 mg/ml). Sample size (*n*): 47, 68, 39, 58. Error bars: median and interquartile range. Note that macrophage chemoattraction mediated by Cxcl11aa and Cxcl11af is abolished by *cxcr3.2* mutation, whereas the chemoattraction mediated by Cxcl11ae is independent of *cxcr3.2*. ns, non-significant; **P<0.01; ***P<0.001.

To assess the chemoattractant properties of the infection-inducible chemokines Cxcl11aa, Cxcl11af and Cxcl11ae, we used *Pichia pastoris* strain *X-33* to express recombinant proteins. As a control, we also expressed zebrafish Cxcl8a (Il8), known to be a potent and neutrophil-specific chemoattractant (Deng et al., 2013; Sarris et al., 2012). All three purified CXCL11-like chemokines showed chemoattractant capabilities towards macrophages when locally injected *in vivo* into the hindbrain at 30 hpf (Fig. 5), whereas no significant macrophage recruitment was exerted by Cxcl8a (supplementary material Fig. S6). Similar levels of these chemokines were injected in the otic vesicle at 54 hpf to evaluate their chemoattractant capabilities towards neutrophils (supplementary material Fig. S6B,C). Cxcl11aa and Cxcl11af did not show chemoattraction of neutrophils under these conditions, whereas Cxcl11ae and Cxcl8a exerted significant neutrophil chemoattraction. To determine whether the macrophage chemoattraction is dependent on *cxcr3.2*, hindbrain injections of the recombinant proteins were performed in both wt and *cxcr3.2* mutants. Both Cxcl11aa and Cxcl11af did not stimulate recruitment upon local injection in *cxcr3.2* mutants when compared with their mock controls (Fig. 5A,C). In contrast, the chemoattraction of phagocytes mediated by Cxcl11ae was independent of *cxcr3.2* mutation (Fig. 5B; supplementary material Fig. S6C). Taken together, these results support a direct ligand-receptor interaction between Cxcr3.2 and the chemokines Cxcl11aa and Cxcl11af that mediates the chemoattraction of macrophages. Differently, Cxcl11ae, which exerted a Cxcr3.2-independent phagocyte chemoattraction, is likely to signal via a yet-unidentified receptor.

### Mutation of *cxcr3.2* affects mycobacterial infection dissemination and granuloma formation

**Fig. 6.**
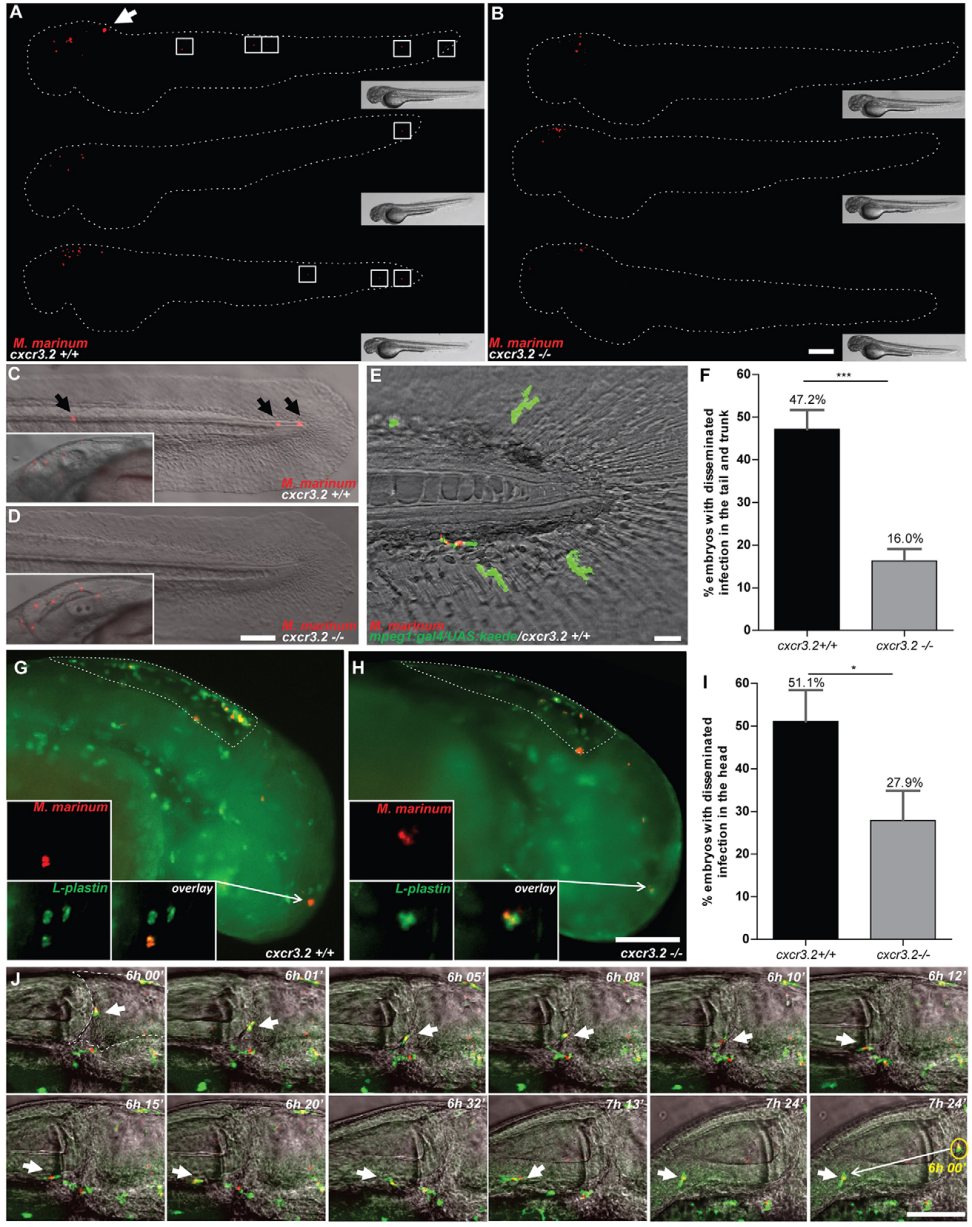
**Effect of *cxcr3.2* mutation on dissemination of local mycobacterial infection within 24 hpi.** (A,B) Representative images of *cxcr3.2^+/+^* and *cxcr3.2*^−/−^ embryos with local and disseminated mycobacterial infection. Embryos were infected at 30 hpf by injecting 200 CFU of *M. marinum* into the hindbrain and images were taken at 24 hpi (54 hpf). In *cxcr3.2^+/+^* embryos, single infected cells are visible distally from the infection (white boxes). Scale bar: 200 μm. (C–E) Details of distal infection emerging from hindbrain infection. The black arrows (C) point at single *M. marinum*-infected cells, present in the tail of a *cxcr3.2^+/+^* fish at 24 hpi but notably absent in the example of a *cxcr3.2^−/−^* fish (D). Particulars of the infected hindbrains of the same embryos are shown in the boxed inserts on the left, indicating similar levels of local infection. Use of the *Tg(mpeg1:gal4/UAS:kaede)* line (E) shows that the infection disseminated from the hindbrain resides in macrophages in *cxcr3.2^+/+^.* Scale bars in C and D: 100 μm; scale bar in E: 20 μm. (F) Quantification of *M. marinum* infection dissemination in the trunk and tail in *cxcr3.2^+/+^* and *cxcr3.2^−/−^* embryos. Embryos were scored positive for dissemination if one or more infected macrophages were observed in the trunk or tail region. The graph demonstrates a significant difference in the total percentage of embryos showing infection dissemination at 24 hpi. Data were accumulated from three independent experiments. Sample size (*n*): 125, 172. Error bars: mean±s.e.m. (G–I) Quantification of *M. marinum* infection dissemination in the head in *cxcr3.2^+/+^* and *cxcr3.2^−/−^* embryos. Representative figures of *cxcr3.2* wild-type (wt; G) and mutant (H) embryos and quantification (I) of dissemination in the head at 6 hours post *M. marinum* infection in the hindbrain. Embryos were scored positive for dissemination if one or more infected macrophages were observed outside the hindbrain limits (dotted line). Insets in G and H show details of infected macrophages outside the hindbrain. The graph in I demonstrates a significant difference in the total percentage of embryos showing infection dissemination at this time point. Arrows in the figures point at the particular of a disseminated infection. Sample size (*n*): 47, 43. Error bars: mean±s.e.m. Scale bar: 200 μm. (J) Time course of a *M. marinum*-infected macrophage egressing from the hindbrain. The image sequence (taken from a *cxcr3.2^+/+^* embryo) represents over a time course of ~1.5 hours that macrophages (green) can facilitate the dissemination of *M. marinum* (red) that is locally delivered in the hindbrain ventricle. The dashed line in the first image represents the hindbrain limits and arrow points at an infected macrophages adhering to the hindbrain boundary. In the subsequent images the arrow points at the infected macrophage egressing from the hindbrain. The yellow circle in the last image of the sequence contains an inset of the infected macrophage from the first image to represent its initial position (6 hpi) compared with its position at the end of the image sequence (~7.5 hpi). Scale bar: 100 μm. *P<0.05; ***P<0.001.

**Fig. 7.**
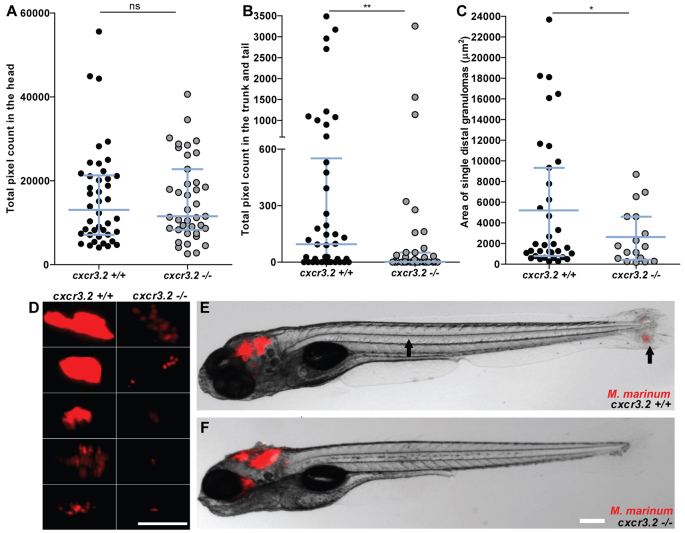
**Effect of *cxcr3.2* mutation on dissemination of local mycobacterial infection at 5 dpi.** (A,B) Quantification of total levels of infection burden in the head and in the trunk and tail of 6 dpf zebrafish larvae. Embryos were injected at 30 hpf in the hindbrain ventricle with 200 CFU of *M. marinum*. Comparable levels of infection are reached locally in the head (A), but disseminated infection burden in the trunk and tail was significantly reduced in *cxcr3.2^−/−^* larvae (B). Data were accumulated from two independent experiments. Sample size (*n*): 42, 39. Error bars: median and interquartile range. (C,D) Size and morphology of distal granulomas in *cxcr3.2^+/+^* and *cxcr3.2*^−/−^ larvae. Distal bacterial clusters that originated occasionally in *cxcr3.2*^−/−^ embryos appeared generally smaller than the ones more frequently formed in the *cxcr3.2^+/+^* siblings. Size was determined by fluorescent bacterial quantification of single distant granulomas in *cxcr3.2^+/+^* and *cxcr3.2^−/−^* (C) and five representative images of each are shown (D). Data were accumulated from two independent experiments. Sample size (*n*): 35 and 17 distal granulomas from 42 and 39 observed *cxcr3.2^+/+^* and *cxcr3.2^−/−^* embryos, respectively. Scale bar: 200 μm. Error bars: median (A,B) or mean (C) and interquartile range. (E,F) Late effects of distal infection emerging from hindbrain infection. Representative images of *cxcr3.2^+/+^* (E) and *cxcr3.2^−/−^* (F) embryos at 5 dpi. Black arrows point at distal dissemination foci in the *cxcr3.2^+/+^* larva. Scale bar: 200 μm. ns, non-significant; *P<0.05; **P<0.01.

**Fig. 8.**
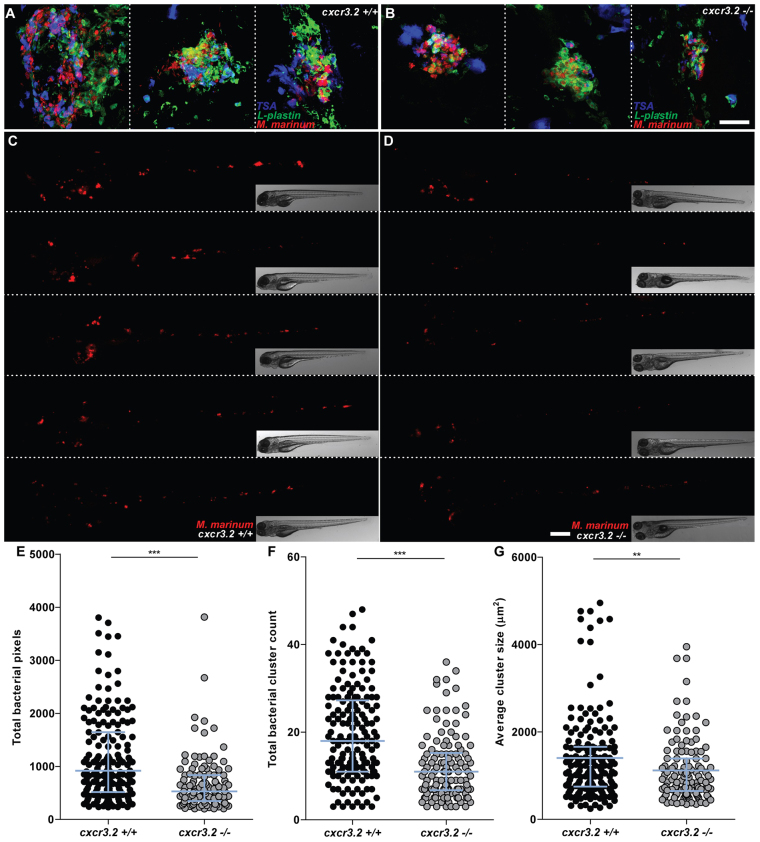
**Effect of *cxcr3.2* mutation on granuloma formation following systemic mycobacterial infection.** (A–D) Representative images of granulomas in systemically infected *cxcr3.2^+/+^* and *cxcr3.2*^−/−^ larvae. Embryos were systemically infected at 1 dpf, injecting 200 CFU of *M. marinum* into the caudal vein. Images of three representative granuloma-like structures of wild-type (wt; A) or mutant (B) larvae were taken on samples fixed at 6 dpf and simultaneously stained for Lp and Mpx (TSA stain) to distinguish macrophages (Lp-positive, TSA-negative) from neutrophils (TSA-positive). Images of representative *cxcr3.2^+/+^* (C) or *cxcr3.2^−/−^* (D) larvae were acquired live at 6 dpf. Scale bar in A,B: 40 μm. Scale bar in C,D: 200 μm. (E–G) Quantification of the impact of *cxcr3.2* mutation on mycobacterial granuloma formation. Total infection burden (total infection fluorescent pixels; E), total number of bacterial clusters (F) and average area of bacterial clusters (G) are significantly reduced under *cxcr3.2*-deficient conditions. Data were accumulated from three independent experiments. Sample size (*n*): 170, 122. Error bars: median (E,F) or mean (G) and interquartile range. **P<0.01; ***P<0.001.

Pathogenic mycobacteria have the ability to resist intracellular macrophage digestion and they can use the macrophages as a vector for distal dissemination of the infection (Clay et al., 2007). We hypothesized that *cxcr3.2* depletion, preventing a high level of macrophage accumulation to the local infection site, might also prevent extensive dissemination and help to locally restrict the infection. To test this hypothesis we followed *M. marinum* hindbrain infection for 24 hours and evaluated the frequency of infection dissemination in *cxcr3.2^+/+^* and *cxcr3.2^−/−^* zebrafish embryos. At 24 hpi, almost 50% of the wt embryos displayed dissemination of the infection from the head to the trunk and tail, whereas, in more than 80% of the mutants, the infection remained locally confined (Fig. 6A–F). Dissemination to other areas of the head could be seen already as early as 6 hpi and also this phenotype was attenuated in *cxcr3.2^−/−^* embryos (Fig. 6G–I). Disseminated bacteria outside the hindbrain and/or midbrain were residing in phagocytes and, in time course experiments, we could visualize that egression of mycobacteria from the ventricles is facilitated by macrophages (Fig. 6J), in agreement with previously published results (Clay et al., 2007). When dissemination to the tail and trunk occurred, one to five dissemination foci could be detected in the *cxcr3.2^+/+^* embryos, whereas *cxcr3.2^−/−^* embryos never showed more than one or two bacterial clusters distally from the original injection point. At 5 dpi, the bacterial burden in the hindbrain was similar between wt and mutant larvae, but mutants still showed lower levels of dissemination of the infection towards distal areas (Fig. 7A,B). The infection foci generated distally developed into typical granuloma-like aggregates, as previously described for the zebrafish-*M. marinum* model (Davis et al., 2002). The size of these granulomatous lesions was significantly reduced in the *cxcr3.2^−/−^* mutant larvae (Fig. 7C–F). Therefore, we concluded that *cxcr3.2*-mediated signaling strongly influences the dynamics of the infection progression and of granuloma formation. To further investigate the relevance of *cxcr3.2* signaling in the formation of granulomas, we injected 200 CFU of *M. marinum* systemically in 1 dpf embryos via the caudal vein, which, in the wt leads to many granulomatous lesions (Clay et al., 2007). Images of single granulomas at 5 dpi, stained for both macrophages and neutrophils, revealed that granuloma-like aggregates could still be formed in *cxcr3.2* mutants. Similar structures and phagocyte compositions were observed when lesions of similar sizes in wt and mutant were compared (Fig. 8A,B). However, it must be noted that a large variation in granuloma structure and composition already exists when comparing different granulomas within the same larva or between different wild types, and this makes it very difficult to assess the effect of a mutation on the general architecture of the granulomas. Despite this, we observed that Cxcr3.2 deficiency provided partial protection against mycobacterial infection. Not only did mutants exhibit reduced levels of infection burden (Fig. 8C–E), but also a reduced number of bacterial clusters (Fig. 8F) and smaller average bacterial cluster size (Fig. 8G). Taken together with the results of hindbrain infection, these data demonstrate the important role of Cxcr3.2-dependent signaling in guiding macrophage-mycobacteria interactions, and show how this signaling leads to direct effects on the infection progression.

## DISCUSSION

The chemokine receptor CXCR3 and its ligands play important roles in the pathogenesis of infectious diseases, autoimmune disorders and cancer (Bondar et al., 2014; Chakravarty et al., 2007; Cohen et al., 2013; Cuenca et al., 2011; Kakuta et al., 2012; Kawada et al., 2007; Oghumu et al., 2014; Pan et al., 2006; Rosas et al., 2005; Seiler et al., 2003; Slütter et al., 2013). In this study, we report on the function of CXCR3 signaling in macrophage recruitment to infection foci and in the early establishment of mycobacterial granulomas. We found that the Cxcr3.2 receptor, one of the three zebrafish homologs of human CXCR3, interacts with infection-inducible zebrafish homologs of the CXCL11 ligand family and is required for the mobilization of macrophages to different pathogens, such as locally delivered *M. marinum* or *S. typhimurium*. Furthermore, mutation of *cxcr3.2* reduced the macrophage-mediated dissemination of *M. marinum*, leading to attenuation of the formation and expansion of granulomatous lesions in both local and systemic models of mycobacterial infection.

CXCR3 is best known as a canonical marker for Th1 cells, but several recent studies have raised interest in the expression of this receptor by macrophages. These studies have implicated CXCR3 signaling in processes as diverse as the recruitment of macrophages to allografts (Kakuta et al., 2012), the macrophage-mediated remodeling of blood vessels (Zhou et al., 2010) and the polarization of macrophages towards an M2 phenotype that promotes tumor progression (Oghumu et al., 2014). Furthermore, CXCR3 signaling has been shown to play a crucial role in the murine neonatal response to sepsis (Cuenca et al., 2011). Like murine neonates, zebrafish embryos and early larvae rely heavily on their innate immune system for defense against infection. During zebrafish embryogenesis, macrophages are the first leukocyte cell type to develop and they express *cxcr3.2* from day 1 (Zakrzewska et al., 2010). In mutants of *cxcr3.2*, or in wt embryos treated with a human CXCR3 antagonist (NBI74330), we observed a significant reduction in the recruitment of macrophages to local bacterial infection in the hindbrain. In contrast, Cxcr3.2-deficient macrophages were able to normally migrate in response to chemically induced wounding or towards Cxcr3.2-independent chemoattractants, such as LTB_4_ and fMLP. These data suggest that Cxcr3.2 signaling is specifically activated by pathogen-induced chemokine signals. We considered a cluster of CXCL11-like chemokines as the putative ligands of Cxcr3.2 and confirmed that two of these, Cxcl11aa and Cxcl11af, exerted chemoattractant activity on macrophages following hindbrain injection of the recombinant proteins. Most likely, Cxcl11ag, which is near-identical to Cxcl11af, also signals through Cxcr3.2. It is currently unknown whether the *cxcl11* genes in zebrafish are IFN-γ-inducible like their mammalian counterparts, but IFN-γ responsive elements are present in the promoters of these genes (van der Aa et al., 2012). In addition, because we were unable to detect expression of the zebrafish *cxcl11* genes *in situ*, the cell types producing these chemokines remain to be established. However, qRT-PCR showed rapid upregulation of *cxcl11aa* and *cxcl11af/ag* gene expression following infection, supporting their function as the ligands mediating the infection-dependent recruitment of Cxcr3.2-positive macrophages.

Expression analysis on FACS-sorted phagocyte populations showed that also *cxcr3.3* is expressed in macrophages, but macrophage motility and recruitment defects in the *cxcr3.2* mutant line indicates that expression of *cxcr3.3* cannot compensate for the loss of function of *cxcr3.2*. In addition, the expression analysis revealed that also neutrophils express *cxcr3.2* at 2 and 6 dpf. Injection of Cxcl11aa or Cxcl11af into the otic vesicle at 2 dpf did not chemoattract a higher number of neutrophils within 3 hours than mock injections, whereas comparable concentrations of these chemokines were able to recruit macrophages into the hindbrain, and comparable concentrations of Cxcl8a and Cxcl11ae mobilized neutrophils when delivered in the otic vesicle. Different explanations can be given for this effect. Firstly, it is possible that different concentrations of chemokines are required to efficiently chemoattract different cell types. Secondly, the requirement of co- stimulatory signals or cell-specific co-receptors might be different between the phagocyte populations. Thirdly, although macrophages and neutrophils at 2 dpf seem to express comparable levels of *cxcr3.2* mRNA, it remains unknown whether similar protein levels of Cxcr3.2 are exposed on their membranes. It should be noted that macrophages and their progenitors are marked by *cxcr3.2* expression already at 1 dpf, whereas its expression could not be detected at this time point on neutrophil progenitors (Zakrzewska et al., 2010). In line with this consideration, it is possible that this different timing in messenger expression impacts the protein levels at 2 dpf.

Macrophages are essential for the dissemination of pathogenic mycobacteria and mediate the formation of both primary and secondary granulomas in the zebrafish host following infection with *M. marinum* (Clay et al., 2007; Davis et al., 2002). When *M. marinum* was locally injected into the hindbrain ventricle of 1-day-old embryos, almost half of the embryos exhibited dissemination within 24 hours, where single infected macrophages migrated out of the ventricle and localized distally. In *cxcr3.2* mutants, this dissemination of the infection was significantly reduced, which might be a consequence of the diminished macrophage attraction to the primary infection source or a direct effect on the retromigration ability of *cxcr3.2* mutant macrophages. When the bacteria were injected intravenously, *cxcr3.2* mutation reduced the formation and the expansion of granulomas, thereby attenuating the dissemination of bacteria and the overall burden of systemic infection. This phenotype might be explained by the reduced motility of macrophages in *cxcr3.2* mutants, because it has been shown that early granulomas in zebrafish larvae expand by spreading of the infection to newly recruited macrophages (Davis and Ramakrishnan, 2009). In agreement, the phenotype of *cxcr3.2* mutant larvae resembles those caused by deficiency in other host (*mmp9*) or bacterial (ESAT-6) factors that also impair macrophage recruitment (Volkman et al., 2010; Davis and Ramakrishnan, 2009).

The zebrafish larval tuberculosis model is limited to the study of the initial stages of granuloma formation by macrophages in a context where the adaptive immune system is not yet functional. A beneficial effect of *CXCR3* mutation has also been observed during chronic infection of BALB/c mice with *Mycobacterium tuberculosis* (Chakravarty et al., 2007). In this model the resistance of CXCR3-deficient mice was attributed to the function of *CXCR3* in T-cell priming. Another study using C57BL/6 mice showed that *CXCR3* mutation affected early granuloma formation after aerosol *M. tuberculosis* infection and correlated this with the invasion of polymorphonuclear neutrophils that produce chemokine signaling via CXCR3 (Seiler et al., 2003). Together, the studies in mice and zebrafish models support further investigation of the CXCR3 signaling axis as a host therapeutic target for tuberculosis. Our study is the first to implicate this signaling axis in macrophage responses that drive the initiation and expansion of mycobacterial granulomas. In future work it will therefore be of great interest to investigate how macrophage and T-cell responses determined by CXCR3 signaling cooperate in the control of mycobacterial infections, using adult zebrafish or mammalian models of tuberculosis.

Recently, another chemokine receptor, Ccr2, has also been shown to mediate macrophage recruitment following hindbrain infection of *M. marinum* in zebrafish embryos (Cambier et al., 2014). This Ccr2-mediated pathway is dependent on the presence of phenolic glycolipids on the mycobacterial cell surface and it recruits a population of macrophages that are permissive for mycobacterial growth, because activation of the host immune response is largely avoided owing to the presence of other cell surface lipids in virulent mycobacteria (phthiocerol dimycoceroserate lipids), which physically mask the underlying PAMPs. *M. marinum* bacteria lacking phenolic glycolipids were still able to recruit macrophages, and morpholino knockdown of either Ccr2 or its ligand Ccl2 attenuated recruitment but did not fully abolish it. These observations indicate that redundant and/or synergistic mechanisms are cooperating in macrophage mobilization. Combined experiments will be necessary to reveal whether the Ccr2-Ccl2 axis is (partially) redundant or synergistic with the Cxcr3-Cxcl11-mediated macrophage recruitment shown here.

Interestingly, we found that Cxcr3.2 is also involved in the basal motility of macrophages under physiological conditions. We hypothesize that the lower basal motility of macrophages in *cxcr3.2* mutants could be due to the inability to sense small amounts of Cxcr3.2 ligands secreted in the macrophage microenvironment. Possibly, the macrophages themselves could be involved in an autocrine or paracrine secretion of these ligands. Similar mechanisms acting via CXCR3 signaling have already been described in the literature. Keratinocytes have been shown to express CXCL10 and CXCR3 to guide their own migration for re-epithelialization in a wound-healing response (Kroeze et al., 2012). Similarly, synovial fibroblasts use this ligand-receptor pair to regulate their invasion of joints in rheumatoid arthritis (Laragione et al., 2011). Furthermore, myeloid cells and hematopoietic progenitors secrete many different chemokines, including CXCR3 ligands, to regulate hematopoiesis in an autocrine or paracrine manner (Majka et al., 2001). The autocrine or paracrine production of Cxcr3.2 ligands could potentially work as a local macrophage stimulator, which might significantly contribute to the surveillance activities that macrophages exert in tissues. During mycobacterial disease, similar mechanisms might be stimulated within the core of the granulomatous lesions, and could be involved in the in-and-out trafficking properties of macrophages, characteristic of these dynamic structures (Chensue, 2013; Fuller et al., 2003). In various animal models of tuberculosis, including the most clinically relevant macaque model, abundant expression of CXCR3 ligands is detected in the core and in the direct neighborhood of the granulomatous lesions (Aly et al., 2007; Fuller et al., 2003; Khader et al., 2009). Our study suggests that this is relevant not only for the recruitment of T cells, but also for regulating macrophage activities in the immunopathology of the granulomatous lesion. A number of studies with selective agonists or antagonists of CXCR3 have already shown beneficial effects on inflammation-associated diseases (O’Boyle et al., 2012; van Wanrooij et al., 2008), and the zebrafish model might be a suitable model to test their effectiveness on mycobacterial infections.

Concluding, here we propose a dual biological role of Cxcr3.2-Cxcl11aa/af ligand-receptor signaling. First, our results implicate Cxcr3.2 and its ligands in surveillance against pathogens by promoting the random patrolling of inactive macrophages. Second, we show that this pathway is involved in the mobilization of macrophages during infection. Depending on the specific interactions of different pathogens with their hosts, a Cxcr3.2-dependent response could be beneficial for the resolution of infection or have an unfavorable effect because, on the one hand, it can sustain the recruitment of macrophages to the infection site, but, on the other hand, it can promote the dissemination of bacteria, as in the case of mycobacterial infection.

## MATERIALS AND METHODS

### Zebrafish lines and maintenance

Zebrafish lines were handled in compliance with the local animal welfare regulations and maintained according to standard protocols (zfin.org). The breeding of adult fish was approved by the local animal welfare committee (DEC) of the University of Leiden (license number: 10612) and adhered to the international guidelines specified by the EU Animal Protection Directive 2010/63/EU. Adult zebrafish were not sacrificed for this study. All experiments in this study were performed on embryos/larvae before the free-feeding stage and did not fall under animal experimentation law according to the EU Animal Protection Directive 2010/63/EU.

Fish lines used in this work were the following: wild-type (wt) strain AB/TL, double-transgenic line *Tg(mpeg1:mcherryF/mpx:eGFP)* (Bernut et al., 2014; Renshaw et al., 2006), homozygous mutant (*cxcr3.2^−/−^*) and wt siblings (*cxcr3.2^+/+^*) of *cxcr3.2^hu6044^*, *Tg(mpeg1:gal4-VP16/UAS-E1b:kaede)*, in short referred to as *Tg(mpeg1:gal4/UAS:kaede)* (Ellett et al., 2011), and the combination of *Tg(mpeg1:gal4/UAS:kaede)* with the *cxcr3.2* mutant strain. The *cxcr3.2^hu6044^* allele was identified by sequencing of an ENU (N-ethyl-N-nitrosourea)-mutagenized zebrafish library and was obtained from the Hubrecht Laboratory and the Sanger Institute Zebrafish Mutation Resource. Heterozygous F2 carriers were outcrossed twice against wt, and were subsequently incrossed. Resulting *cxcr3.2^−/−^* and *cxcr3.2^+/+^* siblings were raised and used to obtain embryos for all the experiments. The combined mutant-transgenic line *Tg(mpeg1:gal4/UAS:kaede/cxcr3.2^−/−^* or *cxcr3.2^+/+^)* were obtained by crossing heterozygous carriers with the original transgenic line and subsequently incrossing the heterozygous offspring. For genotyping, genomic DNA was amplified using forward primer 5′-GGCATCTTTTTTGTTACAGCCTACAGCTTA-3′ and reverse primer 5′-TGGCGATATCGGCGGATAACA-3′, amplifying a 201 base pair (bp) product containing the mutation. The forward primer introduces an additional base change, which only in combination with the mutant allele generates the consensus for *Dde*I restriction enzyme. Therefore, the mutant allele was distinguished from the wt by specific digestion into a 174 fragment that can be separated from the undigested wt amplicon on a 2.5% agarose gel. Alternatively, genotyping was performed by KASP assay using the primers 5′-CATCATAGGAAGTACTGTTGTAGTCA-3′, 5′-CATCATAGGAAGTACTGTTGTAGTCC-3′ and 5′-GGCATCTTTTTTGTTACAGCCTACAGATT-3′. Robustness of both methods was verified several times by sequencing of the amplicons.

Embryos were grown at 28.5°C in egg water (60 μg/ml sea salt, Sera marin, Heinsberg, Germany). For live-imaging or injection assays, larvae were anesthetized in egg water medium containing 0.02% buffered Tricaine (3-aminobenzoic acid ethyl ester; Sigma-Aldrich, St Louis, MO, USA). To prevent melanization, larvae were generally maintained in egg water supplemented with 0.003% PTU (1-phenyl-2-thiourea; Sigma-Aldrich).

### Sequencing

Sequencing of the full coding sequence of *cxcr3.1*, *cxcr3.2* and *cxcr3.3* was obtained by amplification with primers described in supplementary material Table S3. For *cxcr3.2* and *cxcr3.3*, both genomic and cDNA templates extracted from pools of 15–20 embryos were used. Amplification was performed with Phusion high-fidelity DNA polymerase (Thermo-Scientific, Pittsburgh, PA, USA). DNA amplicons were then gel-extracted on 1.5% agarose and column-purified with PureLink quick gel extraction and PCR purification kit (Invitrogen, Life Technologies, Carlsbad, CA, USA). Sequencing with M13Fw, M13Rv universal primers (incorporated in the amplification primers) or with custom-made primers was outsourced to Baseclear (Leiden, The Netherlands). For *cxcr3.1*, sequencing results derive exclusively from genomic DNA amplifications. Amplification of cDNA templates for *cxcr3.2* resulted in a band of identical size in mutant, wt and AB/TL, thereby excluding altered exon/intron arrangements attributable to the ENU-mutagenesis per se or to the *cxcr3.2^hu6044^* allele.

### Bacterial cultures and infection delivery

Approximately 200 CFU (1 nl) of *M. marinum* strain Mma20 expressing mCherry (van der Sar et al., 2004), or *Salmonella enterica* serovar Typhimurium (*S. typhimurium*) strain SL1027 expressing DsRed (van der Sar et al., 2003), were grown and harvested as described previously (Benard et al., 2012; Cui et al., 2011). Embryos were staged at 30 hpf and bacteria or mock control [phosphate buffer saline (PBS) supplemented with 0.1% phenol red (Sigma-Aldrich) and 2% polyvinylpyrrolidone-40 (Sigma-Aldrich)] were locally injected in the hindbrain cavity as described previously (Benard et al., 2012; Cui et al., 2011). Injections of bacteria in the otic vesicle, as shown in Fig. 3, were performed either at 3 dpf (Fig. 3B,I–L) or at 4 dpf (Fig. 3C–H). When infection was delivered systemically, the same dose was instead injected in the caudal vein as in Benard et al. (Benard et al., 2012). As a control, the same dose was spotted onto plates, incubated and counted. Embryos were kept into fresh PTU egg water, incubated at 28.5°C, and collected for qRT-PCR or used for imaging at 1–6 dpf. In Fig. 8A,B, embryos were fixed at 6 dpf in 4% paraformaldehyde in PBSTx (1× PBS supplemented with 0.8% Triton X-100; Sigma-Aldrich) and prepared for Myeloperoxidase (Mpx) activity stain with TSA staining kit (PerkinElmer Inc., Waltham, MA, USA), followed by immunostaining against the pan-leukocyte marker Leukocyte-plastin (Lp) as described previously (Cui et al., 2011).

### FACS-sorting, RNA isolation and qRT-PCR

*Mpeg1:mcherryF*-positive, *mpx:eGFP*-positive and unlabeled cells were sorted from *Tg(mpeg1:mcherryF/mpx:eGFP)* at 2 and 6 dpf. FACS-protocol and RNA isolation were performed according to Rougeot et al. (Rougeot et al., 2014). To evaluate the induction of the *cxcl11* genes upon infections, pools of 18–20 embryos were collected for RNA isolation, snap-frozen in liquid nitrogen and subsequently stored at −80°C. RNA was extracted using Qiazol reagent (Qiagen, Valencia, CA, USA) according to the manufacturer’s guidelines. Residual genomic DNA was removed by DNA-free kit (Ambion, Life Technologies). The cDNA was prepared using the iScript cDNA-synthesis kit (Invitrogen, Life Technologies) and was used as a template for qRT-PCR reaction with iQ SYBR Green Supermix according to the manufacturer’s instructions (Bio-Rad Laboratories, Munich, Germany). Specificity of the amplification reaction was analyzed using dissociation curves. Each qRT-PCR was performed in technical duplicate and on biological replicates as indicated in the figure legends. Reference genes were *eif4a1b* or *eif5* (*eukaryotic translation initiation factor 4a isoform 1b* or *5*) for FACS-sorted cells and *ppiab* (*peptidylprolyl isomerase ab/cyclophilin a*) for infection experiments. Fold changes were determined using the ΔΔ comparative threshold method. Primers are reported in supplementary material Table S3.

### Production of recombinant chemokines and local injections

Synthetic coding sequences for Cxcl11aa, Cxcl11af, Cxcl11ae and Cxcl8a (included as negative control) were generated (Baseclear) according to database accessions (supplementary material Table S2). To enable secretion in yeast, the sequences were codon optimized and the predicted zebrafish signal peptide was replaced with yeast alpha-factor secretion signal, as a result from cloning into pPICZα expression vector (Invitrogen, Life Technologies). Additionally, a HA (human influenza hemagglutinin)-tag was added at the C-terminus to facilitate the purification process and identification. The recombinant chemokines were produced by *Pichia pastoris* strain *X-33* transformed with the chemokine vectors as described previously (Wu and Letchworth, 2004). Proteins were purified via Fast Protein Liquid Chromatography in NaCl salt gradient and finally desalted and concentrated by membrane filtrations on Amicon Ultra Centrifugal filter devices with a nominal molecular weight limit of 3 kilodaltons (Amicon, Merck KGaA, Ireland), using 50 mM sodium phosphate buffer pH 6.5 as a washing and suspension vehicle. Purity and identity of the proteins were confirmed by trypsinization and electrospray mass-spectrometry. The recombinant chemokines (0.5–1.5 mg/ml), LTB_4_ (leukotriene B4; Santa Cruz Biotechnology, Santa Cruz, CA, USA; 10.1 ng/ml), fMLP (N-formyl-methionyl-leucyl-phenylalanine; Sigma-Aldrich; 0.2 mg/ml) or mocks [sodium phosphate buffer pH 6.5 for the chemokines, 5% DMSO (Sigma-Aldrich) in PBS for fMLP and 0.02% ethanol (Sigma-Aldrich) in PBS for LTB_4_] were supplemented with 0.1% phenol red and injected at 30 hpf in the hindbrain ventricle (1 nl) or at 52 hpf in the otic vesicle (0.5 nl) as described previously (Benard et al., 2012; Cui et al., 2011). In both cases, embryos were fixed at 3 hpi in 4% paraformaldehyde in PBSTx and prepared for Lp immunostaining as in Cui et al. (Cui et al., 2011). At 30 hpf, the population of fully differentiated leukocytes is represented almost exclusively by macrophages (Henry et al., 2013; Lieschke et al., 2001); thereby, we could assume that nearly all the Lp-stained cells able to migrate and infiltrate in the ventricle represented macrophages at this developmental stage. As is shown in supplementary material Fig. S3, only one to two *mpx*-positive cells [*mpx*-whole mount *in situ* hybridization as in reference (Cui et al., 2011)] could be counted within the perimeter of the hindbrain in this experimental setting at 3 hours post local bacterial infection, which is less than 10% of the cells positive for the macrophage marker *mfap4*. At later developmental stages, in order to discern between neutrophils and macrophages, samples were processed also with a neutrophil-specific Mpx activity staining as described previously (Cui et al., 2011), by using the leukocyte peroxidase (Myeloperoxidase) staining kit (Sigma-Aldrich) for the histochemical detection of the enzymatic activity of Mpx. Leukocytes accumulated at the injected cavity (macrophages: Lp-positive and Mpx-negative; neutrophils: Mpx-positive) were counted using a Leica MZ16FA fluorescence stereomicroscope (Leica Microsystems, Rijswijk, The Netherlands).

### Chemically induced (ChIn) inflammation assay

3-dpf larvae were exposed to 10 μM copper sulphate (CuSO_4_; Sigma-Aldrich) for 2 hours as described previously (d’Alençon et al., 2010). Treated larvae were then fixed and used for combined Mpx activity staining and Lp immunostaining as described above.

### Pharmacological treatment with NBI74330

Bath-treatment with the CXCR3 high-affinity antagonist NBI74330 or vehicle treatment (0.5% DMSO) was started at 27 hpf by exposing dechorionated embryos to 50 μM of the drug in medium. Embryos were incubated for 3 hours at 28.5°C and then injected in the hindbrain with mock or *M. marinum* as described above. Injected embryos were maintained for an additional 3 hours in 50 μM NBI74330 or vehicle alone and then fixed in 4% paraformaldehyde/PBSTx and prepared for Lp immunostaining as described previously (Cui et al., 2011).

### Imaging and image quantification

Fixed or live embryos and larvae were imaged using a Leica MZ16FA fluorescence stereomicroscope. For time-lapse experiments, samples were mounted in 2% low-melting-point agarose (SphaeroQ, Burgos, Spain) and images were acquired with a laser-scanning confocal microscope (Leica TCS SPE, Leica Microsystems or Zeiss Observer 6.5.32, Carl Zeiss, Sliedrecht, The Netherlands). To assess the average speed of macrophages (Fig. 1J,K), a time-lapse experiment was performed and quantification was obtained on overlaid z-stacks by Fiji/ImageJ software (NIH, Bethesda, MD, USA) using the ManualTrack plug-in as described elsewhere (Meijering et al., 2012). The average speed was calculated as the average of all the speeds assumed by every single macrophage at each time point. Analysis was performed by cumulating three experiments in which 15–21 macrophages per embryo were followed. To quantify the morphological differences between macrophages in *cxcr3.2* mutants and wt, bacteria were injected into the otic vesicle of *Tg(mpeg1:gal4/UAS:kaede/cxcr3.2^+/+^* or *cxcr3.2^−/−^)* larvae at 3 dpf and fixed at 4 hpi. Images of macrophages were acquired in the trunk. Perimeter and area of the cells were obtained by Fiji/ImageJ using the Analyze Particles plug-in. The circularity index (CI) corresponding to each cell was obtained by the formula: CI=4π (area/perimeter^2^), resulting in an index that ranges from 0 (infinitely branched structure) to 1 (perfect circle). Macrophages were classified in five different intervals of circularity based on their CI (0.0 to 0.19, 0.2 to 0.39, 0.4 to 0.59, 0.6 to 0.79, 0.8 to 1.0) and the average percentages of macrophages in each interval were estimated for *cxcr3.2^+/+^* or *cxcr3.2^−/−^* (Fig. 3I). To estimate the divergence of distribution of *cxcr3.2^+/+^* and *cxcr3.2^−/−^* macrophages from the overall mean, the percentages in each interval were divided by the average percentage of mutants and wt assumed in that interval, using the formulas: Deviation_(_*_cxcr3.2_*_+/+)_=%*cxcr3.2*^+/+^/[(%*cxcr3.2*^+/+^ + %*cxcr3.2*^−/−^)/2] and Deviation_(_*_cxcr3.2−/−_*_)_=%*cxcr3.2*^−/−^/[(%*cxcr3.2*^+/+^ + %*cxcr3.2*^−/−^)/2] (Fig. 3J). To quantify the dissemination of bacterial infection (Fig. 6F,I), the presence or absence of infection distally from the infected site was evaluated, giving a score of 1 in case of dissemination and a score of 0 in case of absent dissemination. Quantification of total bacterial pixels (Fig. 7A,B and Fig. 8E) was obtained using dedicated bacterial pixel count program as in Stoop et al. (Stoop et al., 2011). Total bacterial cluster count (Fig. 8F) was performed manually from images. Quantification of the area of single distal clusters (Fig. 7C) and average area of disseminated granulomas (Fig. 8G) were performed using ImageJ quantification tools as in Elks et al. (Elks et al., 2013).

### Statistical analysis

In the survival test (Fig. 1E), non-significant deviation from Mendelian rate was evaluated by χ^2^ test on four independent replicates. For qRT-PCR, statistical significance was estimated on five (Fig. 1A–C), four (Fig. 4A,B) or three (Fig. 4C,D) biological replicates by two-tailed *t*-tests on ln(n)-transformed relative induction folds. All the other experiments were statistically analyzed using GraphPad Prism 4 or 5 (GraphPad Software, La Jolla, CA, USA). Where correction for non-parametric distribution was required (Fig. 1F,G; Fig. 2; Fig. 3A,B; Fig. 3M; Fig. 5; Fig. 6; Fig. 7A,B; Fig. 8E,F), comparisons between two groups were performed with two-tailed Mann-Whitney test and comparisons among more than two groups were performed with Kruskal-Wallis test, followed by Dunn’s multiple comparison test. When a parametric distribution was assumed (Fig. 1A–C; Fig. 1L; Fig. 3I,J; Fig. 4; Fig. 8G), comparisons between two groups were performed with two-tailed *t*-test. In Fig. 7C, significance was estimated with an unpaired *t*-test with Welch’s correction, suitable to compare parametric data having different variances. Significance (*P*-value) is indicated with: ns, non-significant; *P<0.05; **P<0.01; ***P<0.001.

## Supplementary Material

Supplementary Material
