# Supplementary material for: The CXCR3-CXCL11 signaling axis mediates macrophage recruitment and dissemination of mycobacterial infection
Source: Dis Model Mech. 2015 Jan 8;8(3):253–69. doi: 10.1242/dmm.017756 (PMC4348563; doi:10.1242/dmm.017756)
Supplement: Supplementary Material [file supp_8_3_253__index.html]

The CXCR3-CXCL11 signaling axis mediates macrophage recruitment and dissemination of mycobacterial infection — Supplementary Material 

# The CXCR3-CXCL11 signaling axis mediates macrophage recruitment and dissemination of mycobacterial infection

## DMM017756 Supplementary Material

**Files in this Data Supplement:**

- **Supplementary Material**
