# Supplementary material for: The CXCR3-CXCL11 signaling axis mediates macrophage recruitment and dissemination of mycobacterial infection
Source: Dis Model Mech. 2015 Jan 8;8(3):253–69. doi: 10.1242/dmm.017756 (PMC4348563; doi:10.1242/dmm.017756)
Supplement: Supplementary Material [file supp_8.3.253_DMM017756.pdf]

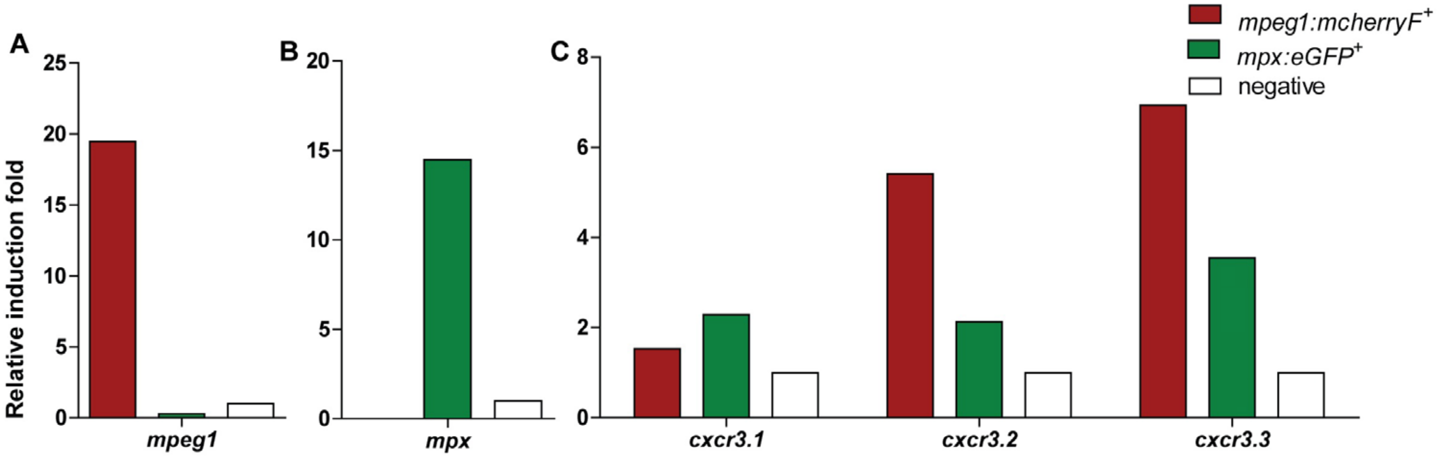

Figure S1: Expression of *cxcr3.2* and its paralogs *cxcr3.1* and *cxcr3.3* in FACS-sorted phagocytes at 6 dpf. Graphs represent the relative induction fold of the macrophage marker *mpeg1* (A), the neutrophil marker *mpx* (B), and of the *cxcr3* paralogs (C) in FACS-sorted macrophages and neutrophils from the combined transgenic line *Tg(mpeg1:mcherryF/mpx:eGFP)* at 6 dpf. Expression of *cxcr3.2* and *cxcr3.3* could be detected in both macrophages and neutrophils, while *cxcr3.3* was not significantly enriched in the FACS-sorted populations when compared to the non-labeled cell fraction. Reference gene: *eif5*.

Figure S2: Protein sequence multiple alignment of human and zebrafish CXCR3 chemokine receptors. Residue color from blue to yellow indicates increasing degree of amino acid conservation. The alignment and the tree were obtained using CLC main workbench 6.8.4 by Neighbour Joining Algorithm. Gap costs were given with a penalty score of 10 for each gap open, and an additional score of 1 per each extension; no cost was associated to end gaps. Extracellular (light green bars), Transmembrane (black bars), and Intracellular (light blue bars) domains were predicted with CLC main workbench 6.8.4. Ligand binding domains (dark green bars) and conserved residues or similar residues within the binding domains (black asterisks) were predicted according to Trotta *et al.*, 2009 (Trotta *et al.*, 2009). The numbers at the tree nodes denote the bootstrap for 10000 replicates. Single alignment of the predicted zebrafish chemokine receptor proteins to the canonical isoform of CXCR3 (hsaCXCR3 isoform 1) were performed with clustalo algorithm (http://www.uniprot.org/align) and provided the following % of identity: dreCXCR3.1 (ENSDARG00000007358): 39.1%, dreCXCR3.2 (ENSDARG00000041041): 35.7%, dreCXCR3.3 (ENSDARG00000070669) isoform 1: 29.8% and dreCXCR3.3 isoform 2: 29.6%.

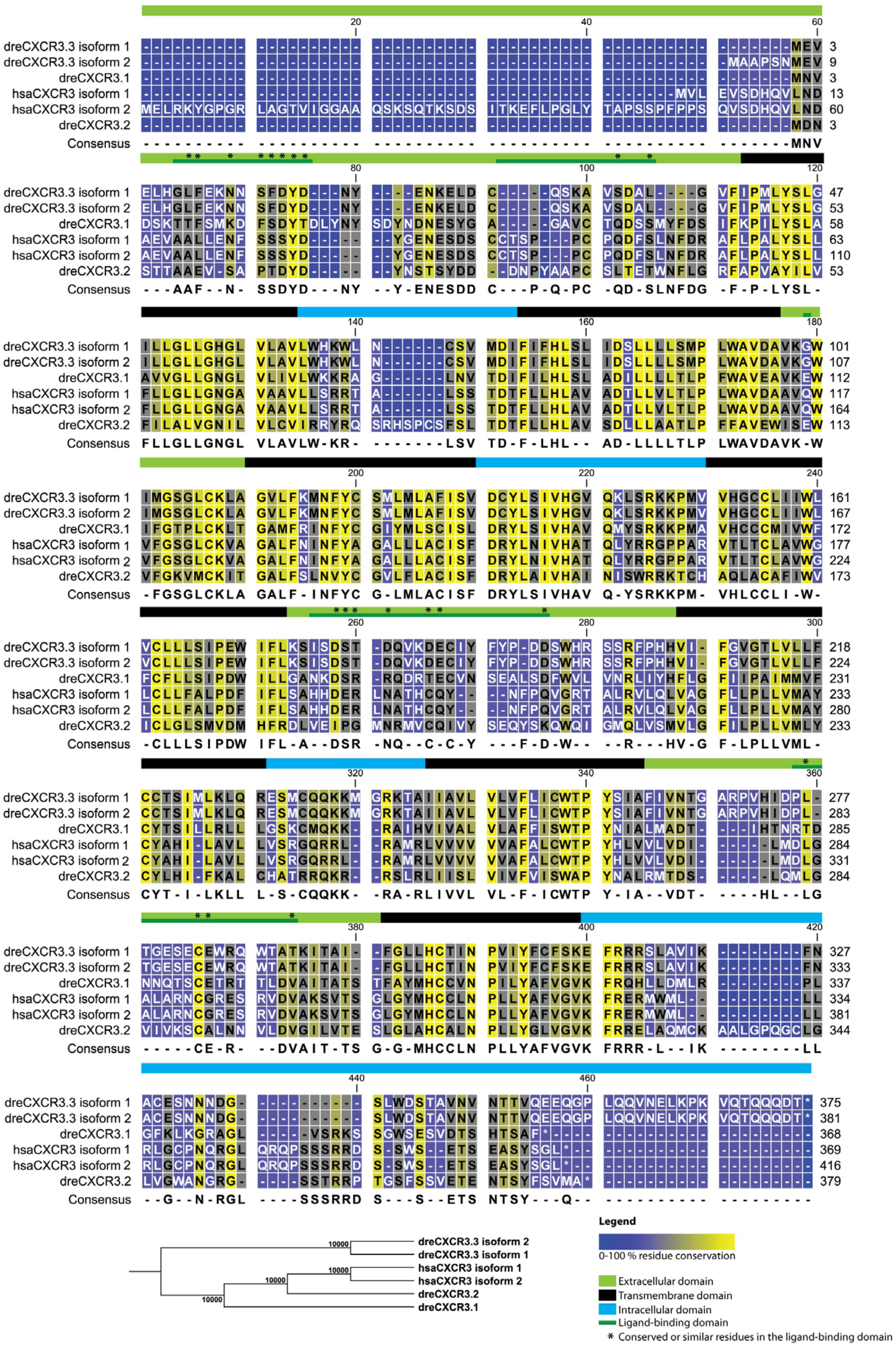
dreCXCR3.3 isoform 1
dreCXCR3.3 isoform 2
dreCXCR3.1
hsaCXCR3 isoform 1
hsaCXCR3 isoform 2
dreCXCR3.2
Consensus
10000
Legend
0-100 % residue conservation
Extracellular domain
Transmembrane domain
Intracellular domain
Ligand-binding domain
* Conserved or similar residues in the ligand-binding domain

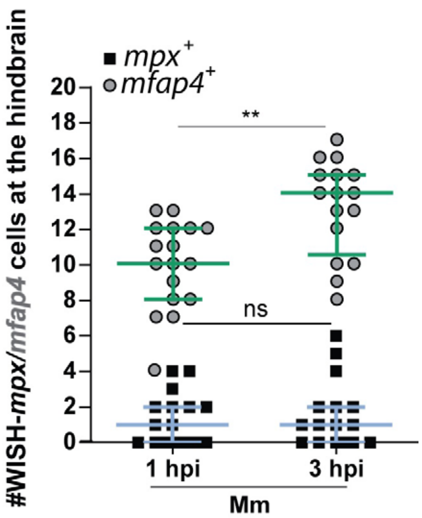

Figure S3: Macrophages are the predominant phagocyte cell type recruited to local hindbrain infection in 31-33 hpf embryos. Embryos were locally injected into the hindbrain cavity at 30 hpf with 100 CFU of *M. marinum* and fixed at 1 and 3 hpi. Double fluorescent *in situ* hybridization was performed with *mfap4* as a macrophage marker and *mpx* as a neutrophil marker (Zakrzewska *et al.*, 2010). The average number of *mfap4*-positive macrophages in the hindbrain at 1 or 3 hpi exceeds the average number of *mpx*-positive neutrophils approximately 10-fold and a significant difference between the time points was observed only for macrophages. Sample size (*n*): 17, 16. Error bars: median and interquartile range. ns, non-significant; **P<0.01.

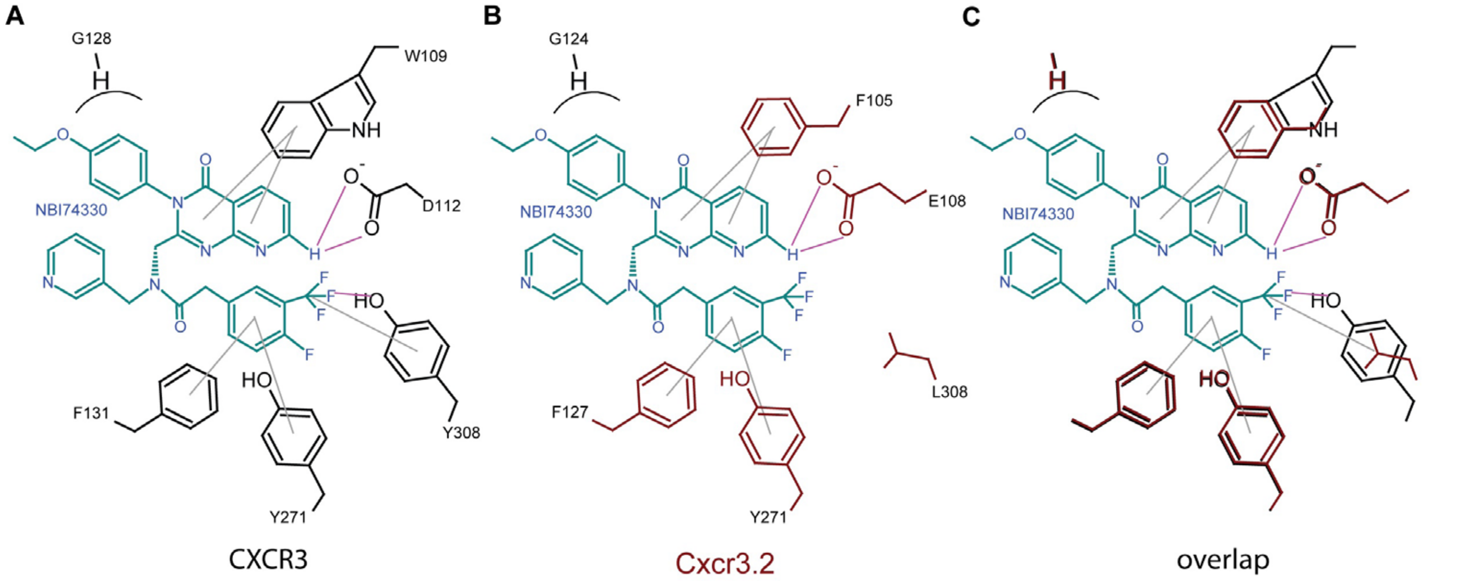

Figure S4: Predicted molecular docking of NBI74330 into the transmembrane minor pocket of Cxcr3.2. The figure represents the high conservation of the transmembrane minor pocket of zebrafish Cxcr3.2 with human CXCR3. Side chains of proposed interacting residues are shown in black for CXCR3 (A) and in red for Cxcr3.2 (B). The ligand NBI74330 is shown in cyan. Suggested receptor/ligand interactions are depicted as colored lines. Polar and hydrogen bonding interactions are shown as pink lines, whereas aromatic interactions are shown as gray lines. C: overlay of the CXCR3 and Cxcr3.2 binding pocket. Prediction of ligand binding for CXCR3 is according to ref. (Scholten et al., 2014). Predictions of ligand binding for Cxcr3.2 is according to sequence alignment and amino acid similarity in the corresponding positions between CXCR3 and Cxcr3.2.

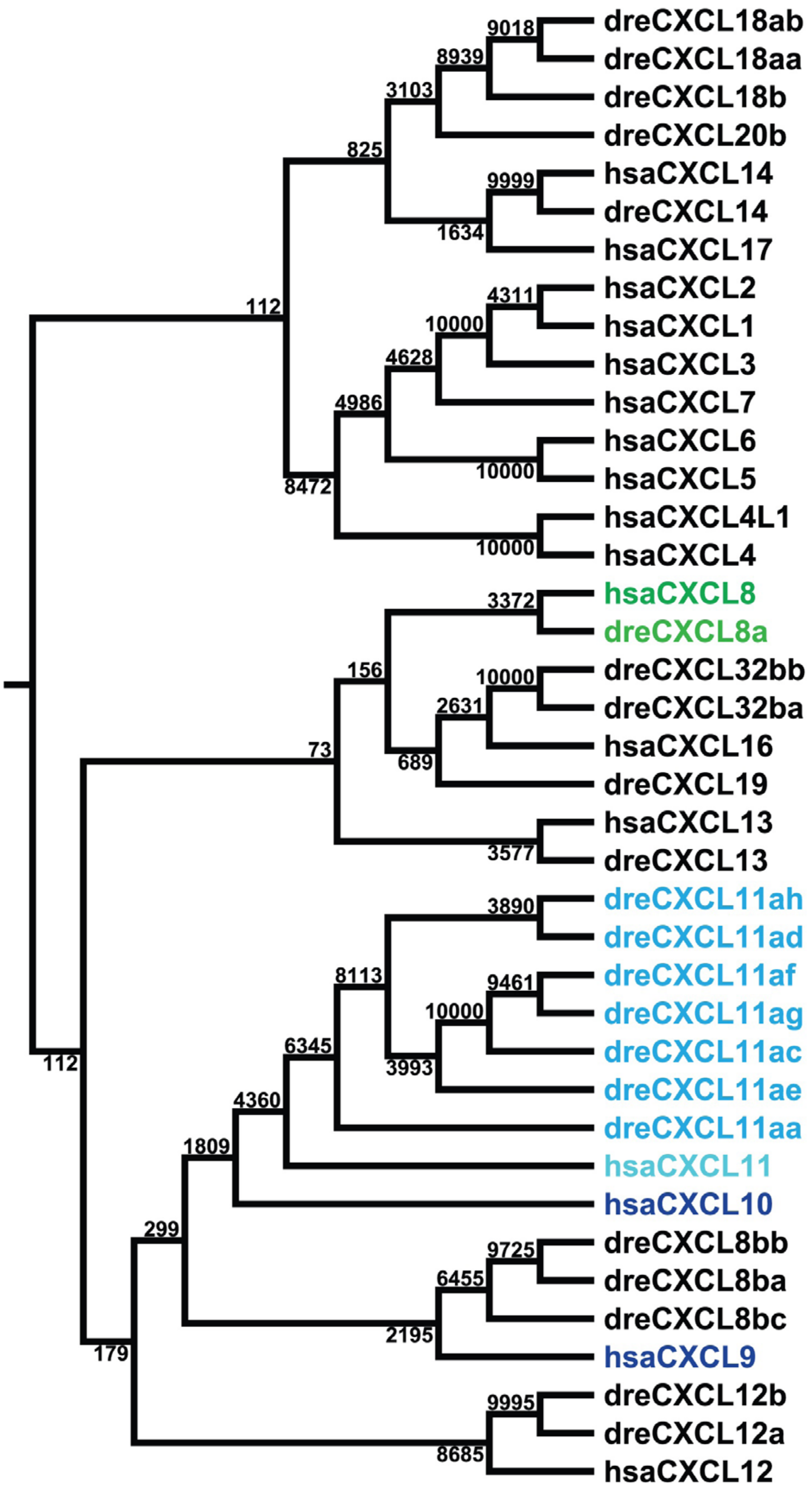

Figure S5: Phylogenetic tree of human and zebrafish chemokine protein sequences. The alignment and the tree were obtained using CLC main workbench 6.8.4 by Neighbour Joining Algorithm. Gap costs were given with a penalty score of 10 for each gap open, and an additional score of 1 per each extension; no cost was associated to end gaps. Additionally, the CXC motif of the chemokine was set as fixed alignment point. The numbers at the tree nodes denote the bootstrap for 10000 replicates. Light blue: Cxcl11-like cluster, containing human CXCL11 and seven zebrafish Cxcl11-like chemokines. Dark blue: human CXCL9 and CXCL10. Green: Cxcl8-like cluster, containing human CXCL8 (IL8) and zebrafish Cxcl8a (Il8). Nomenclature according to ref. (Nomiyama et al., 2013).

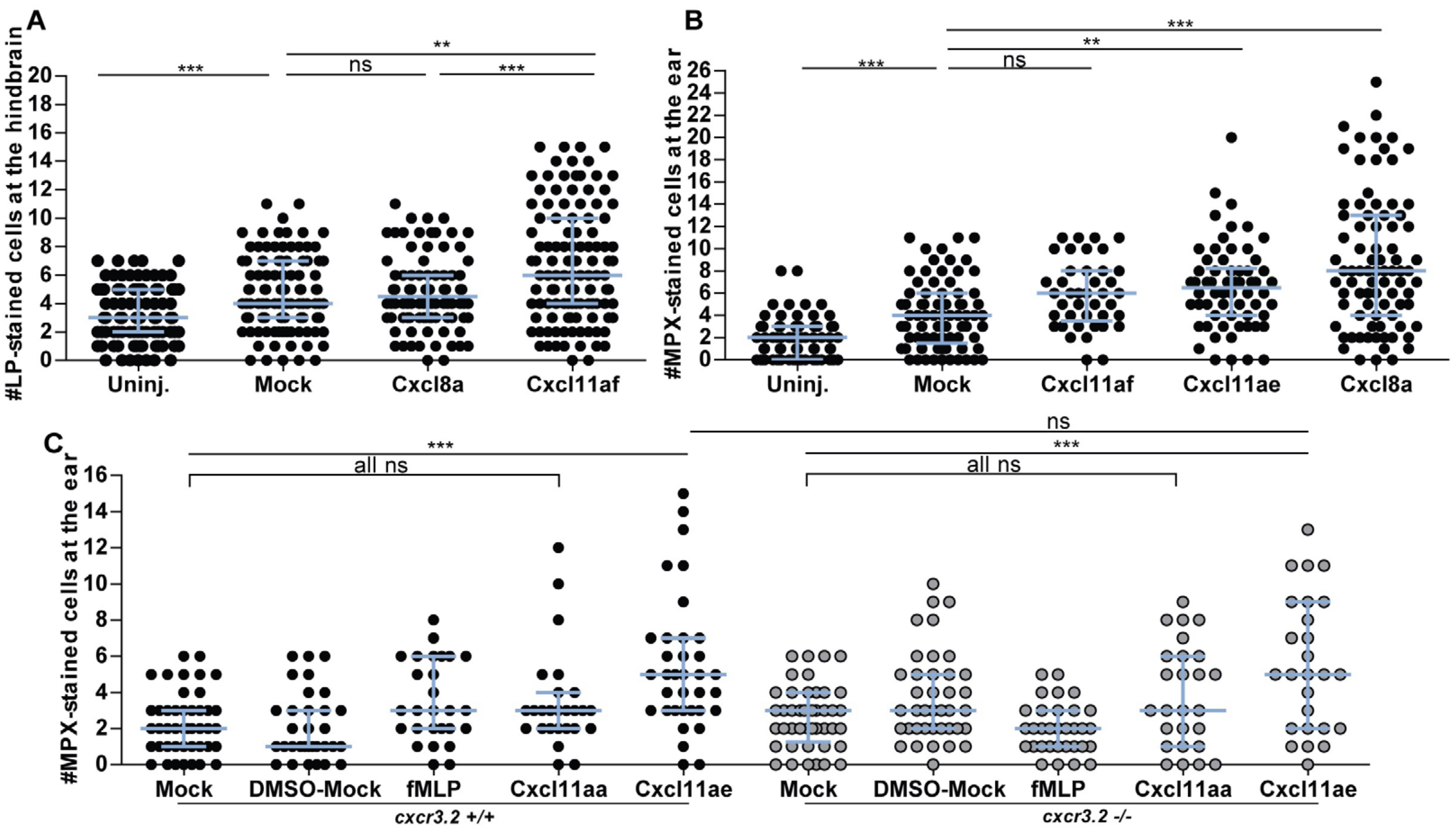

Figure S6: Chemoattractive effects of recombinant chemokines on different phagocytes. (A) Chemoattraction of macrophages to the hindbrain ventricle. Recombinant proteins or buffer (mock) were injected into the hindbrain ventricle of wild type (AB/TL) embryos at 30 hpf and Lp-stained cells accumulating in 3 hours within the hindbrain limits were counted as macrophages. Cxcl11af but not Cxcl8a significantly attracted macrophages. Sample size (*n*): 106, 100, 94, 112. Error bars: median and interquartile range. (B-C) Chemoattraction of neutrophils to the otic vesicle. Recombinant proteins or mock were injected into the otic vesicle of wild type (AB/TL) embryos (B) or *cxcr3.2*$^{+/+}$ and *cxcr3.2*$^{-/-}$ siblings (C) at 54 hpf and neutrophils accumulating in 3 hours within the otic vesicle were counted after Mpx activity staining. Neutrophil attraction by recombinant Cxcl8a (Il8) (B) was in agreement with previous reports (Deng et al., 2013). Note that Cxcl11ae significantly recruited neutrophils in all zebrafish lines, while Cxcl11af (B), Cxcl11aa (C), and fMLP (C) did not exert significant neutrophil attraction above mock injections. Sample size (n) in B: 75, 77, 41, 62, 83. Sample size (*n*) in C: 44, 32, 27, 26, 35, 44, 39, 32, 26, 26. Error bars: median and interquartile range. ns, non-significant; **P<0.01; ***P<0.001.

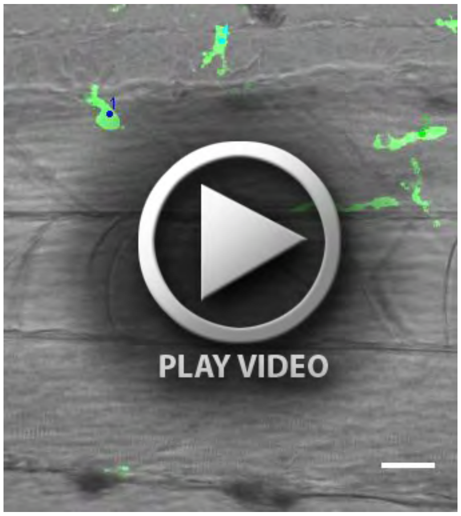

Movie S1. Macrophage basal motility in *cxcr3.2*$^{+/+}$ embryos. Paths of 5 representative macrophages of the trunk. Macrophages were followed contemporary for 3 hours and confocal time lapse images were taken every 6 minutes. The paths were followed and analyzed using Image J ManualTrack plugin. *cxcr3.2*$^{-/-}$ macrophages (compare with supplementary material Movie S2) have a lower basal movement capability than *cxcr3.2*$^{+/+}$ macrophages. See also Fig. 1J-L. Scale bar depicted in the first photogram: 20 µm.

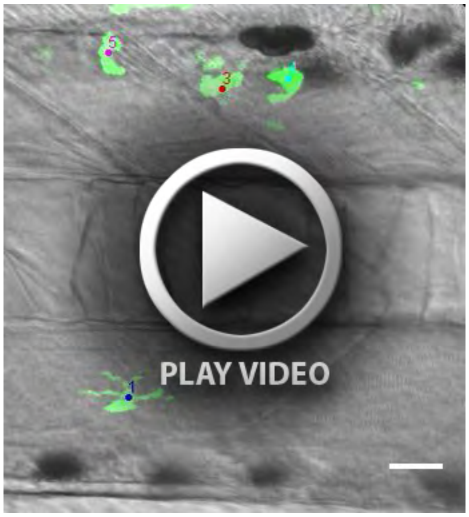

Movie S2. Macrophage basal motility in *cxcr3.2*$^{-/-}$ embryos. Paths of 5 representative macrophages of the trunk. Macrophages were followed contemporary for 3 hours and confocal time lapse images were taken every 6 minutes. The paths were followed and analyzed using Image J ManualTrack plugin. *cxcr3.2*$^{-/-}$ macrophages have a lower basal movement capability than *cxcr3.2*$^{+/+}$ macrophages (compare with supplementary material Movie S1). See also Fig. 1J-L. Scale bar depicted in the first photogram: 20 µm.

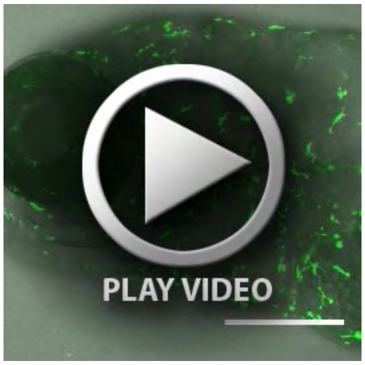

Movie S3. Macrophage recruitment following *M. marinum* infection in the otic vesicle in *cxcr3.2* $^{+/+}$ larvae. A *Tg(mpeg1:gal4/ UAS:kaede*) *cxcr3.2*$^{+/+}$ larva was injected with 200 CFU of *M. marinum* into the otic vesicle (dotted line) at 4 dpf. The video represents the situation monitored from 1 to 5 hpi, with acquisition every 5 minutes. See also Fig 3. It should be noted that since acquisitions start at 1 hpi, a difference in macrophage number in the surrounding of the otic vesicle is already present between mutants and wt. Scale bar depicted in the first photogram: 250 µm.

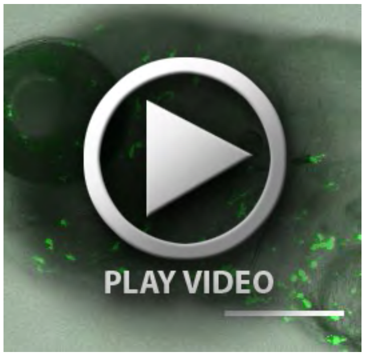

Movie S4. Macrophage recruitment following *M. marinum* infection in the otic vesicle in *cxcr3.2*$^{-/-}$ larvae. A *Tg(mpeg1:gal4/UAS:kaede*) *cxcr3.2*$^{-/-}$ larva was injected with 200 CFU of *M. marinum* in the otic vesicle (dotted line) at 4 dpf. The video represents the situation monitored from 1 to 5 hpi, with acquisition every 5 minutes. See also Fig 3. It should be noted that since acquisitions start at 1 hpi, a difference in macrophage number in the surrounding of the otic vesicle is already present between mutants and wt. Scale bar depicted in the first photogram: 250 µm

Protein	Amino acid position	Strain	Variant	PROVEAN score	Predicted effect
Cxcr3.1	203	*Reference**	E	0.000	Neutral
		AB/TL	E/K	-0,945	Neutral
		cxcr3.2$^{+/+}$	E/K	-0,945	Neutral
		cxcr3.2$^{-/-}$	E	0.000	Neutral
Cxcr3.1	292	*Reference*	E	0.000	Neutral
		AB/TL	E/K	-1.803	Neutral
		cxcr3.2$^{+/+}$	E	0.000	Neutral
		cxcr3.2$^{-/-}$	K	-1.803	Neutral
Cxcr3.1	351	*Reference*	K	0.000	Neutral
		AB/TL	R	0.645	Neutral
		cxcr3.2$^{+/+}$	R	0.645	Neutral
		cxcr3.2$^{-/-}$	R	0.645	Neutral
Cxcr3.2	16	*Reference*	Y	0.000	Neutral
		AB/TL	Y	0.000	Neutral
		cxcr3.2$^{+/+}$	Y	0.000	Neutral
		cxcr3.2$^{-/-}$	STOP	***cxcr3.2***^{hu6044}	Non-sense
Cxcr3.2	93	*Reference*	V	0.000	Neutral
		AB/TL	I	0.048	Neutral
		cxcr3.2$^{+/+}$	I	0.048	Neutral
		cxcr3.2$^{-/-}$	I	0.048	Neutral
Cxcr3.3	48	*Reference*	M	0.000	Neutral
		AB/TL	L	0.332	Neutral
		cxcr3.2$^{+/+}$	L	0.332	Neutral
		cxcr3.2$^{-/-}$	L	0.332	Neutral
Cxcr3.3	75	*Reference*	C	0.000	Neutral
		AB/TL	C/P	-0.547	Neutral
		cxcr3.2$^{+/+}$	C/P	-0.547	Neutral
		cxcr3.2$^{-/-}$	C	0.000	Neutral
Cxcr3.3	95	*Reference*	S	0.000	Neutral
		AB/TL	T	2.931	Neutral
		cxcr3.2$^{+/+}$	T	2.931	Neutral
		cxcr3.2$^{-/-}$	T	2.931	Neutral
Cxcr3.3	236	*Reference*	E	0.000	Neutral
		AB/TL	E/D	-0.328	Neutral
		cxcr3.2$^{+/+}$	E/D	-0.328	Neutral
		cxcr3.2$^{-/-}$	E	0.000	Neutral
Cxcr3.3	238	*Reference*	M	0.000	Neutral
		AB/TL	M/L	-0.611	Neutral
		cxcr3.2$^{+/+}$	M/L	-0.611	Neutral
		cxcr3.2$^{-/-}$	M	0.000	Neutral
Cxcr3.3	244	*Reference*	M	0.000	Neutral
		AB/TL	M/I	-0.473	Neutral
		cxcr3.2$^{+/+}$	M/I	-0.473	Neutral
		cxcr3.2$^{-/-}$	I	-0.473	Neutral
Cxcr3.3	297	*Reference*	T	0.000	Neutral
		AB/TL	S	-0.463	Neutral
		cxcr3.2$^{+/+}$	S	-0.463	Neutral
		cxcr3.2$^{-/-}$	S	-0.463	Neutral
Cxcr3.3	327	*Reference*	L	0.000	Neutral
		AB/TL	L/Q	-2.562	Neutral
		cxcr3.2$^{+/+}$	L/Q	-2.562	Neutral
		cxcr3.2$^{-/-}$	Q	-2.562	Neutral
Cxcr3.3	346	*Reference*	D	0.000	Neutral
		AB/TL	D/E	0.098	Neutral
		cxcr3.2$^{+/+}$	D/E	0.098	Neutral
		cxcr3.2$^{-/-}$	D	0.000	Neutral
Cxcr3.3	350	*Reference*	V	0.000	Neutral
		AB/TL	V/E/D	0.895/1.275	Neutral
		cxcr3.2$^{+/+}$	V/E/D	0.895/1.275	Neutral
		cxcr3.2$^{-/-}$	D	0.895	Neutral
Cxcr3.3	378	*Reference*	Q	0.000	Neutral
		AB/TL	Q/E	-0.077	Neutral
		cxcr3.2$^{+/+}$	Q/E	-0.077	Neutral
		cxcr3.2$^{-/-}$	E	-0.077	Neutral

**Reference*: according to ENSEMBL accessions

Table S1: Non-synonymous single nucleotide polymorphisms in *cxcr3.1*, *cxcr3.2,* and *cxcr3.3* coding sequences. Several non-synonymous single nucleotide polymorphisms (nsSNPs) are present in *cxcr3.1*, *cxcr3.2*, and *cxcr3.3* genes that are inherited in association with the ENU-mutagenized *cxcr*3.2^{hu6044} allele. Except for the Cxcr3.1 E292K variant, all the nsSNPs linked to the *cxcr3.2*$^{-/-}$ fish did not differ from the ones represented also in the *cxcr3.2*$^{+/+}$ fish. Furthermore, all nsSNPs, including the Cxcr3.1 E292K variant, were detected also in the AB/TL wt strain. Analysis using the PROVEAN software tool (Protein Variation Effect Analyzer, http://provean.jcvi.org) (Kumar et al, 2009; Choi et al., 2012), predicts that the nsSNPs are unlikely to have an impact on the protein functionality. The threshold to score the possibility of protein non functionality was set to -4.1 (Choi et al., 2012). Note: Amino acid notations for Cxcr3.3 are represented accordingly to their position in Cxcr3.3 splicing isoform 2. No additional amino acid replacements were specifically found in the splicing isoform 1.

Name	Gene Accession	Protein Sequence
hsaCXCL9	ENSG00000138755	mkksgvlfllgiillvligvqgTPVVRKGRCSCISTNQGTIHLQSLKDLKQFAPSPSCEKIEIIATLKN<br>GVQTCLNPDSADVKELIKKWEKQVSQKKKQKNGKKHQKKKVLKVRKSQRSRQKKTT
hsaCXCL10	ENSG00000169245	mnqtailicclifltlsgiqgVPLSRTVRCTCISISNQPVNPRSLEKLEIIPASQFCPRVEIIATMKKK<br>GEKRCLNPESKAIKNLLKAVSKERSKRSP
hsaCXCL11	ENSG00000169248	msvkgmaialavilcatvvqgFPMFKRGRCLCIGPGVKAVKVADIEKASIMYPSNNCDKIEVIIT<br>LKENKGQRCLNPKSKQARLIIKKVERKNF
dreCxcl11aa	Gene ID: 798892	mktvtalllvslavvaiegQHMKSQRCVCLGAGLNMVKPVLIEKIEILPSSPSCGHMEVIATLKN<br>GAGKRCLNPKSKFTKKIIDKIEKNNRNAR
dreCxcl11ac	GeneID:100334604	mktlaaflltcliagkvngQDNTSRARCFCADKGINMVLLKNIEKVEIFPPSPSCNKNEIVVTLK<br>NGAGQKCLNPDSKFTQNVVLKAIGKRMQQSVPHSTTTGTVKSSMTSSTSAPTAFK
dreCxcl11ad	Gene ID: 567656	mktlaavvllgyllvikvegQARAPRSRCLCADKGVNMVSPKLIEKVDIIPPTPSCGNLEIVVTLK<br>NGAEPKCLSPDSKFTQKYLMKALEKRTLQK
dreCxcl11ae	ENSDARG00000092423	mktaaflvflaffifipgQKKFNRCSCVGKGLDRVALRNIEKFEIIHPSPSCGKQEIIVTMKSSEQ<br>KCLNPESKFTQELIRRALEK
dreCxcl11af	ENSDARG00000094706	mktlaaflllscliagevngQDRSSRARCFCVDKGLNMVLLKNLDKVEIFPPSPSCNKHEIVVTL<br>KNGAGQKCLNPDSKFTKNVVLKAIGKRMQQSVPHSTTTGTVKSSMTSSTSAPTAFK
dreCxcl11ag	ENSDARG00000093779	mktlaaflllscliagevngQDRSSRARCFCVDKGLNMVLLKNLEKVEIFPPSPSCNKHEIVVTL<br>KNGAGQKCLNPDSKFTKNVVLKAIGKRMQQSVPHSTTTGTVKSSMTSSTSAPTAFK
dreCxcl11ah	ENSDARG00000095747	mktaaafvalgcflmvevkgKIPDLKNRCLCADKGANNVNLKTIEKIQIIHPSPSCKRLEIVVTLM<br>KGAGKKCLNPESNLGKNILKALRKKKLTAVRRMNPA
dreCxcl8a	Gene ID: 100002946	mtskiisvcvivflafltiiegMSLRGLAVDPRCRCIETESRRIGKHIKSVELFPPSPHCKDLEIIATL<br>MTTGQEICLDPSAPWVKKIIDRIIVNRKP
hsaCXCR3 isoform 1	ENSG00000186810	MVLEVSDHQVLNDAEVAALLENFSSSYDYGENESDSCCTSPPCPQDFSLNFDRAFLP<br>ALYSLLFLLGLLGNGAVAAVLLSRRTALSSTDTFLLHLAVADTLLVLTLPLWAVDAAVQ<br>WVFGSGLCKVAGALFNINFYAGALLLACISFDRYLNIVHATQLYRRGPPARVTLTCLAV<br>WGLCLLFALPDFIFLSAHHDERLNATHCQYNFPQVGRTALRVLQLVAGFLLPLLVMAY<br>CYAHILAVLLVSRGQRRLRAMRLVVVVVVAFALCWTPYHLVVLVDILMDLGALARNCG<br>RESRVDVAKSVTSGLGYMHCCLNPLLYAFVGVKFRERMWMLLLRLGCPNQRGLQRQ<br>PSSSRRDSSWSETSEASYSGL
hsaCXCR3 isoform 2	ENSG00000186810	MELRKYGPGRLAGTVIGGAAQSKSQTKSDSITKEFLPGLYTAPSSPFPPSQVSDHQVL<br>NDAEVAALLENFSSSYDYGENESDSCCTSPPCPQDFSLNFDRAFLPALYSLLFLLGLL<br>GNGAVAAVLLSRRTALSSTDTFLLHLAVADTLLVLTLPLWAVDAAVQWVFGSGLCKVA<br>GALFNINFYAGALLLACISFDRYLNIVHATQLYRRGPPARVTLTCLAVWGLCLLFALPDF<br>IFLSAHHDERLNATHCQYNFPQVGRTALRVLQLVAGFLLPLLVMAYCYAHILAVLLVSR<br>GQRRLRAMRLVVVVVVAFALCWTPYHLVVLVDILMDLGALARNCGRESRVDVAKSVT<br>SGLGYMHCCLNPLLYAFVGVKFRERMWMLLLRLGCPNQRGLQRQPSSSRRDSSWS<br>ETSEASYSGL
dreCxcr3.1	ENSDARG00000007358	MNVDSKTTFSMKDFSDYTDLYNYSDYNDNESYGAGAVCTQDSSMYFDSIFKPILYSLA<br>AVVGLLGNGLVLIVLWKKRAGLNVTDIFILHLSLADILLLLTLPFWAVEAVKEWIFGTPLC<br>KLTGAMFRINFYCGIYMLSCISLDRYLSIVHAVQMYSRKKPMAVHCCCMIVWFFCFLLS<br>IPDWILLGANKDSRRQDRTECVNSEALSDFWVLVNRLIYHFLGFIIPAIMMVFCYTSILL<br>RLLLGSKCMQKKRAIHVIVALVLAFFISWTPYNIALMADTIHTNRTDNNQTSCETRTTLD<br>VAITATSTFAYMHCCVNPILYAFVGVKFRQHLLDMLRPLGFKLKGRAGLVSRKSSGWS<br>ESVDTSHTSAF
dreCxcr3.2	ENSDARG00000041041	MDNSTTAAEVSAPTDYDYNSTSYDDDNPYAAPCSLTETWNFLGRFAPVAYILVFILAL<br>VGNILVLCVIRRYRQSRHSPCSFSLTDTFLLHLAVSDLLLAATLPFFAVEWISEWVFGK<br>VMCKITGALFSLNVYCGVLFLACISFDRYLAIVHAINISWRRKTCHAQLACAFIWVICLGL<br>SMVDMHFRDLVEIPGMNRMVCQIVYSEQYSKQWQIGMQLVSMVLGFILPLLVMLYCY<br>LHIFKALCHATRRQKRRSLRLIISLVIVFVISWAPYNALRMTDSLQMLGVIVKSCALNNV<br>LDVGILVTESLGLAHCALNPLLYGLVGVKFRRELAQMCKAALGPQGCLGLVGWANGR<br>GSSTRRPTGSFSSVETENTSYFSVMA
dreCxcr3.3 isoform 1	ENSDARG00000070669	MEVELHGLFEKNNSFDYDNYENKELDCQSKAVSDALGVFIPMLYSLGILLGLLGHGLV<br>LAVLWHKWLNCSVMDIFIFHLSLIDSLLLLSMPLWAVDAVKGWIMGSGLCKLAGVLFK<br>MNFYCSMLMLAFISVDCYLSIVHGVQKLSRKKPMVVHGCCLIIWLVCLLLSIPEWIFLKS<br>ISDSTDQVKDECIYFYPDDSWHRSSRFPHHVIFGVGTLVLLFCCTSIMLKLQRESMCQ<br>QKKMGRKTAIIAVLVLVFLICWTPYSIAFIVNTGARPVHIDPLTGESECEWRQWTATKIT<br>AIFGLLHCTINPVIYFCFSKEFRRRSLAVIKFNACESNNNDGSLWDSTAVNVNTTVQEE<br>QGPLQQVNELKPKVQTQQQDT
dreCxcr3.3 isoform 2	ENSDARG00000070669	MAAPSNMEVELHGLFEKNNSFDYDNYENKELDCQSKAVSDALGVFIPMLYSLGILLGL<br>LGHGLVLAVLWHKWLNCSVMDIFIFHLSLIDSLLLLSMPLWAVDAVKGWIMGSGLCKL<br>AGVLFKMNFYCSMLMLAFISVDCYLSIVHGVQKLSRKKPMVVHGCCLIIWLVCLLLSIP<br>EWIFLKSISDSTDQVKDECIYFYPDDSWHRSSRFPHHVIFGVGTLVLLFCCTSIMLKLQ<br>RESMCQQKKMGRKTAIIAVLVLVFLICWTPYSIAFIVNTGARPVHIDPLTGESECEWRQ<br>WTATKITAIFGLLHCTINPVIYFCFSKEFRRRSLAVIKFNACESNNNDGSLWDSTAVNV<br>NTTVQEEQGPLQQVNELKPKVQTQQQDT

Table S2. List of chemokines and chemokine receptors used in this work with accession codes and protein sequences. Red letters: amino acids at the splicing sites of the mRNA; blue letters: conserved CXC-chemokine motifs. Part in gray: active chemokine upon removal of the signal peptide

Primers used in qRT-PCR reactions

Gene Name	Gene Accession	Sequence (5'-3')
cxcr3.1	ENSDARG00000007358	**Fw:** CTTTCCTGCATCAGTCTCGACC<br>**Rv:** TGACGTCTGGAGTCCTTGTTGG
cxcr3.2	ENSDARG00000041041	**Fw:** CCTCTGTTGGTAATGCTGTATTGC<br>**Rv:** ACACGATGACTAAGGAGATGATGAG
cxcr3.3	ENSDARG00000070669	**Fw:** GCTCTCAATGCCTCTCTGGG<br>**Rv:** GACAGGTAGCAGTCCACACT
ppiab	ENSDARG00000042247	**Fw:** ACACTGAAACACGGAGGCAAAG<br>**Rv:** CATCCACAACCTTCCCGAACAC
cxcl11aa	Gene ID: 798892	**Fw:** ACTCAACATGGTGAAGCCAGTGCT<br>**Rv:** CTTCAGCGTGGCTATGACTTCCAT
cxcl11ac	Chr5: 44501563-44502267 (-)	**Fw:** TCTGACCTGCCTGATCGCTGGA<br>**Rv:** TGCCTTTGTCAGCACAGAAGCACC
cxcl11ad	Gene ID: 567656	**Fw:** AGGCCAGGCGAGAGCTCCAA<br>**Rv:** TCCACAAGAAGGGGTCGGTGGT
cxcl11ae	ENSDARG00000092423	**Fw:** AGGGTTGCACTGAGGAACATTGAGA<br>**Rv:** AGCCCTCCTGATTAATTCCTGGGT
cxcl11af/ag	ENSDARG00000094706<br>ENSDARG00000093779	**Fw:** GCTGGAGAGGTCAACGGTCAGGA<br>**Rv:** TGCAAGATGGACTCGGAGGGAAGA
cxcl11ah	ENSDARG00000095747	**Fw:** GAGGTGAAAGGCAAAATA<br>**Rv:** TGCTCCTTTATCAGCACACAAACA

Primers used for amplification and sequencing of cxc3.1, cxcr3.2 and cxcr3.3

- Amplification of genomic DNA templates

Gene Name		Sequence (5'-3')
cxcr3.1	Set 1	**Fw:** GTTGTAAAACGACGGCCAGTATGAATGTTGACTCAAAAACAAC<br>**Rv:** CAGGAAACAGCTATGACCTATTATCCAAAAACATTCTTTTCCTCAC
cxcr3.1	Set 2	**Fw:** GTAAAACGACGGCCAGGGGAAACGGCTTGGTTCTGA<br>**Rv:** CAGGAAACAGCTATGACTGTATGCATGAAACAGCAAAGAGAA
cxcr3.1	Set 3	**Fw:** GTAAAACGACGGCCAGGATTTGGAACAACCTTTTTCAGCA<br>**Rv:** CAGGAAACAGCTATGACCTTGAAGCCCAGAGGTCGTA
cxcr3.1	Set 4	**Fw:** GTTGTAAAACGACGGCCAGTCTTCATTTGCATCTTTCTTCATCAG<br>**Rv:** CAGGAAACAGCTATGACCTCAGAAAGCAGATGTGTGGG
cxcr3.2		**Fw:** GTAAAACGACGGCCAGGGCGCCTTATAATGCTCTGC<br>**Rv:** CAGGAAACAGCTATGACCCATGGAAAGCTTCAGTTTTTAC
cxcr3.3	Set 1	**Fw:** GTAAAACGACGGCCAGTTCCCAGCAGATGCGTTACA<br>**Rv:** CAGGAAACAGCTATGACGTGCGCAAGCAAACGTAAAC
cxcr3.3	Set 2	**Fw:** GTAAAACGACGGCCAGGTTCCGCCAACTGCTTTACTTT<br>**Rv:** CAGGAAACAGCTATGACTAAACAGCGTCTTTACCAGATCCC
cxcr3.3	Set 3	**Fw:** GTAAAACGACGGCCAGGACTGCTACCTGTCCATCGTT<br>**Rv:** CAGGAAACAGCTATGACGCTCATGTCTAAATACAGTTTGCTG

- Amplification of cDNA templates

Gene Name	Sequence (5'-3')
cxcr3.2	**Fw:** GTTGTAAAACGACGGCCAGTATGGACAACTCAACAACAGCCGCAG<br>**Rv:** CAGGAAACAGCTATGACCTCAGGCCATGACAGAAAAGTACGAAGTG
cxcr3.3 isoform2 Fw	**Fw:** GTTGTAAAACGACGGCCAGTATGGCAGCACCTTCAAACATGG
cxcr3.3 isoform1 Fw	**Fw:** GTTGTAAAACGACGGCCAGTATGGAGGTAGAGCTTCACGG
cxcr3.3 common Rv	**Rv:** CAGGAAACAGCTATGACCTCATGTATCCTGCTGCTGGG

- Primers used for sequencing

Gene Name	Sequence (5'-3')
M13 universal Fw	**Fw:** GTAAAACGACGGCCAG
M13 universal Rv	**Rv:** CAGGAAACAGCTATGAC
Cxcr3.2 E1 cDNA Rv	Rv: CGCCAGGATAAACACCAG
Cxcr3.3 cDNA Rv	Rv: GGCAGTCCAGTTCTTTGTTC

Table S3. List of q-RTPCR, amplification, and sequencing primers used in this work.

References

Please see the main article for references not listed below:

Trotta, T., Costantini, S. and Colonna, G. (2009). Modelling of the membrane receptor CXCR3 and its complexes with CXCL9, CXCL10 and CXCL11 chemokines: putative target for new drug design. *Mol. Immunol.* **47**, 332-339.
